# Caesarean deliveries and their impact on financial burden and quality of life among post-partum mothers in Tamil Nadu, India

**DOI:** 10.1371/journal.pone.0358778

**Published:** 2026-09-22

**Authors:** Retna Sheeja Pushpa Raj, Prakash Babu Kodali

**Affiliations:** 1 Department of Public Health Sciences, Central University of Kerala, Kasaragod, Kerala, India; 2 Center for Tobacco Control Research and Education, University of California, San Francisco, California, United States of America; Centre for Interdisciplinary Research, Centre of Excellence, INDIA

## Abstract

**Introduction:**

Caesarean section (C-section) deliveries are on the rise globally, often with significant health and economic consequences. This study investigates the prevalence and factors associated with C-section deliveries, and their association with out-of-pocket expenditures (OOPE) and health-related quality of life (HRQL) among post-partum mothers in Tamil Nadu, India.

**Methods:**

A cross sectional study among 475 randomly selected post-partum mothers was conducted during April-June 2023. Data on demographic and obstetric characteristics and OOPE were collected using a structured survey questionnaire. HRQL was assessed using the 12-Item Short Form Survey (SF-12). Binary logistic regression analysis was used to compute Adjusted Odds Ratios (AOR) to identify the factors associated with C-section deliveries, and identify the association between C-section deliveries with OOPE and HRQL.

**Results:**

C-section deliveries accounted for 52.60% of all births. High-risk pregnancies (AOR = 17.21, 95% CI = 7.57–39.13, p < 0.001), and private healthcare facilities (AOR = 3.02, 95% CI = 1.62–5.65, p = 0.001) were associated with C-sections. C-section delivery was significantly associated with higher OOPE (AOR = 2.05, 95% CI = 1.22–3.47, p = 0.007) and poor physical HRQL (AOR = 0.31, 95%CI = 0.18–0.55, p < 0.001).

**Conclusions:**

One in two deliveries in Tamil Nadu are C-section deliveries and were associated with higher financial burden and poorer quality of life among post-partum mothers. Strategies targeted at reducing unwarranted C-sections are needed.

## Introduction

The maternal mortality ratio (MMR) is on a steady decline globally and in India. As of 2018–20, the MMR in India was recorded at 97 per 100,000 live births, a significant decrease from 130 per 100,000 live births in the year 2014–16 [1]. According to the fifth round of National Family Health Survey (NFHS) conducted during 2019–2021, 88.6% of all deliveries in India and 99.6% of all deliveries in Tamil Nadu are institutional deliveries [2]. However, > 90% of mothers undergoing institutional deliveries incurred out of pocket expenses (OOPE) [3]. In five years from 2015–16–2019–21, the OOPE associated with institutional deliveries increased by 26.5% [4,5]. In Tamil Nadu alone, the OOPE associated with institutional deliveries in public hospitals increased by 21% [4,5]. In India, households pay around Indian Rupees (INR).10,000/- per institutional delivery [6]. Also, the cost of delivery is known to vary according to the mode of delivery (i.e., Caesarean Section [C-section]/Vaginal Birth), place of delivery (public vs private), health status of the mother and child, and treatment duration [7]. While the World Health Organization (WHO) recommends the a judicious use of C-section procedure at levels of 10–15% to be optimum to prevent maternal mortality [8]. C-sections account for over 20% of all deliveries in India [2].

Tamil Nadu reported a C-section prevalence of 44.9% in 2019−20, which is substantially higher than the C-section rates in developed economies like the United States (US) and the United Kingdom (UK) [2]. From 2015 to 2021, the prevalence of C-sections in public hospitals in Tamil Nadu increased by 36.9% compared to a 24.4% relative increase in the private hospitals [5]. C-section rates are strongly associated with place of delivery, social norms, high risk pregnancies, and fertility preferences [9,10]. In addition to incurring higher costs, mothers undergoing C-section have poorer health related quality of life (HRQL) [11]. A longitudinal study from Australia reported that women who delivered by C-section had poorer physical and mental health scores [12]. Another study from New Delhi, India reported that women undergoing C-section had significantly poorer scores across the domains of physical functioning, role physical, bodily pain, vitality, social functioning and mental health [13].

Previous studies conducted based on the National Family Health Surveys (NFHS) 2015−16 (round 4) and 2019−21 (round 5) established the association between C-section deliveries and high out of pocket expenses (OOPE), and a high odds of financial distress among households of women undergoing C-section in private healthcare facilities [7,14]. However, these studies were primarily focussed on OOPE estimates. Evidence on the role of high-risk pregnancies and female sterilization as indications for C-section remains to be studied, specifically in the contexts like Tamil Nadu with a high prevalence of high-risk pregnancies, close to 100% institutional deliveries, fertility rates below replacement levels, and a focussed policy target to reduce MMR. Moreover, earlier studies comparing C-section and Vaginal deliveries predominantly focussed on OOPE, while other economic aspects such as wage loss to family caregiver and financial distress involving borrowings or sale of assets to pay OOPE were not studied. While there a few studies on C-section’s impact on HRQL, they are also limited by the volume of evidence in Indian settings, small sample size, and predominant focus on mothers during the post-natal period. We conducted this study (i) to assess the prevalence and factors associated with C-section deliveries in Tamil Nadu, and (ii) compare the out-of-pocket expenditures and health related quality of life among mothers undergoing C-section and vaginal deliveries.

## Materials and methods

### Study design and study setting

We conducted a community based cross-sectional survey of post-partum mothers from 15^th^ April 2023–30^th^ June 2023. The survey was conducted among a randomly sampled mothers from three districts (i.e., Kanyakumari, Tuticorin, and Pudukkottai) of Tamil Nadu. These three districts were selected to represent three distinct multidimensional poverty index(MPI) levels (i.e., low, moderate, and high respectively) and had C-section prevalence ranging from 43.1% to 67.6% [4,15].

### Sampling

The sample size was estimated using OpenEpi version 3.01 (https://www.openepi.com/SampleSize/SSPropor.htm) based on the formula


$$\begin{array}{c} \text{n}=\left[\text{DEFF}*\text{Np}(\text{1}-\text{p})\right]/\left[{\text{d}}^{\text{2}}/{\text{Z}}_{\text{1}-\alpha /\text{2}}^{\text{2}}*(\text{N}-\text{1})+\text{p}*(\text{1}-\text{p})\right] \end{array}$$

An initial sample of 384 was estimated at an anticipated prevalence (p) of C-section deliveries of 50%, with confidence level at 95%, an absolute precision (d) of 5%, and a design effect (DEFF) of 1.0. Further, considering a non-response rate of 20% the sample was inflated to 461, and rounded to a final sample size of 475 post-partum women.

Sampling was conducted employing multi-stage stratified cluster random sampling approach. Given that maternal healthcare utilization is impacted by socio-economic and development indicators [6,7,16], we used the MPI levels to categorize the districts in Tamil Nadu into low, moderate, and high based on the MPI India report published by NITI Aayog, Government of India [15]. MPI is a composite index of 12 indicators across three dimensions (Health [including Nutrition, Child & adolescent mortality, Maternal Health], Education, and Standard of Living) capturing deprivations that individuals/households experience simultaneously [15]. One district from each MPI group were randomly sampled as the primary sampling units. Each district was further stratified into urban municipalities, and rural panchayats. From the list of the primary health centers (PHCs) in the district, one PHC from the urban municipalities and one PHC from the rural panchayats were randomly sampled resulting in six clusters [3 urban and 3 rural]. From each sampled PHC, the birth registries were accessed and line-list of institutional births between 1^st^ April 2022–31^st^ March 2023 was prepared. From the line-list of births, 77–81 post-partum mothers per PHC were randomly sampled using a computer programme. A total of 156, 160, and 159 mothers were sampled from Kanyakumari, Tuticorin, and Pudukkottai districts respectively. Each potential participant was approached and screened for inclusion. Eligibility criteria included mothers aged 18 years and above who had an institutional delivery and whose most recent childbirth occurred between 1 April 2022 and 31 March 2023. Mothers with self-reported mental health conditions were excluded. In addition, women who had experienced a stillbirth or infant death, as well as those who did not provide consent, were considered ineligible for inclusion in the study. A total of 510 mothers were approached to attain the sample size of 475, with most [n = 34] of the excluded mothers refusing consent to participate.

### Data collection

The survey was conducted between 15^th^ April 2023–30^th^ June 2023. Caesarean section was captured as a self-reported response to the item “*With respect to the birth of your recent child, what was the mode of delivery?”*. The items for obstetric history included gravida, place of antenatal care (ANC) visits, episiotomy during delivery, and female sterilization. Further, the survey questionnaire also captured details on the indications for high-risk pregnancy and intrapartum complications. The indications for high-risk pregnancy included severe anaemia, preeclampsia, gestational diabetes, hypothyroidism, young primigravida/elderly gravida, twin/multiple pregnancy, malpresentation, previous C-section delivery, placenta previa, previous bad obstetric history, Rh negative, short stature, obesity, and history of systemic illness. A mother was considered to have a high-risk pregnancy if she reported to have at least one of these indications during her pregnancy. The intrapartum complications included breech presentation, prolonged labour, and excessive bleeding. The OOPE incurred towards institutional delivery was captured as a total of indirect costs incurred towards transportation, paid-caregivers, food & accommodation; and direct costs including OOPE for hospital stay, tests, medicines and other expenses. The data on mode of delivery and obstetric history were captured based on self-report and verified by mother and child protection card and/or hospital discharge summery. The data on OOPE was primarily self-reported.

The HRQL was measured using a 12-Item Short Form Health Survey (SF-12) questionnaire developed for the Medical Outcomes Study [17]. It captures HRQL under eight domains *“i) physical functioning, ii) role-physical, iii) bodily pain, iv) general health perceptions, v) vitality, vi) social functioning, vii) role-emotional, viii) general mental health”*, with the former four capturing the physical component summary (PCS) and later four capturing the mental component summary (MCS) [17]. SF-12 has been shown to have a significant internal consistency and structural validity to measure HRQL among post-partum mothers [18]. In this study, SF-12 had an acceptable internal reliability with Cronbach’s alpha (α) measured at 0.85. The questionnaire was translated into vernacular version (Tamil), and back translated to English to check for the consistency of translations. Translations and back translation of the questionnaire was done by the first author a native speaker ensuring to account for cultural nuances in translated versions of the questionnaire. Further, the translated versions were checked with another native speaker not part of study team to ensure consistency of interpretation. Content and face validity of the questionnaire was established prior to undertaking the study through a review of the questionnaire by the research team, and two independent experts. The survey was conducted in Tamil, and data were collected as self-reported responses.

### Data cleaning and analysis

The data entry, cleaning, and analysis was conducted using International Business Machine’s (IBM) Statistical Package for Social Sciences (SPSS) version 27 and Stata version 13 (StataCorp, College Station, TX, USA). Based on a prior analysis of NFHS-4 which reported the mean OOPE for institutional deliveries in Tamil Nadu as 10,063 [19], and NFHS-5 estimates of average cost of institutional deliveries in Indian settings [7], we categorized the OOPE paid towards institutional deliveries into two categories ≤INR.10000/- (approximately 120 USD), and>INR.10000/-. The HRQL variables, i.e., PCS & MCS were computed as continuous scores with higher PCS & MCS scores representing better HQRL in physical and mental health components respectively. The PCS and MCS questions of SF-12 were coded following the standardized procedures, by clearing the out-of-range values, followed by reverse coding of four negative questions (SF1, SF8, SF9, SF10), creating the indicator items, applying specific weights, and finally standardizing the PCS and MCS scores by using constants [17,20]. The PCS and MCS scores were transformed to categorical variables with a PCS & MCS score of 50 and above being categorized as good, and below 50 was categorized as poor [17,21,22].

We computed the percentage prevalence and 95% confidence interval (CI) of the C-section deliveries, and indications for high-risk pregnencies. A descriptive comparison of the indications of high-risk pregnancies and intrapartum complications among vaginal and C-section deliveries were made. The descriptive estimates and bivariate comparisons of OOPE for delivery, cost of postnatal care and treatment, wage loss to family caregiver, amount received as financial assistance, financial distress, and HRQL variables of PCS & MCS between the vaginal and C-section deliveries was undertaken. The Mann-Whitney U test and Chi-Square test were used to assess the statistical significance of the difference in these estimates by mode of delivery. We developed logistic regression models to (i) identify the factors associated with C-section deliveries, (ii) ascertain the association of C-section deliveries with out-of-pocket expenditure, (iii) assess the association of mode of delivery with physical component summery of HRQL, and (iv) on the mental component summery of HRQL. Given the dependent variables in each of the regression models were dichotomous, we used binary logistic regression. The independent variables comprised of socio-demographic characteristics (i.e., district, place of residence, wealth index, and education), obstetric history (high risk pregnancy, intra partum complications, gravida), place of delivery, and mode of delivery. These variables were included based on prior published evidence on institutional deliveries [12,13,19,23–27], and bivariate associations observed in the data. Adjusted odds ratios (AOR) were computed to adjust for the confounding effect of independent variables in the regression models. Variance inflation factor (VIF) was computed to assess for multicollinearity between independent variables, and a VIF of up to 3.00 was considered acceptable. Area under receiver operating characteristic curves (AUROC) were calculated to evaluate the discriminative ability of the logistic regression models. Sensitivity analyses were performed using clustered robust standard errors, excluding women with previous C-sections from analysis, and by log transformation of OOPE as outcome variable (S1 File, Tables 4–7).

### Ethics statement

We conducted the study complying with the relevant ethical guidelines including the Declaration of Helsinki. The Institutional Human Ethics Committee of the Central University of Kerala (Ref.No. IHEC/CUK/2023/23) approved the study. Additionally, permissions to collect data were obtained from the Director of Public Health and Preventive Medicine, Tamil Nadu. Informed consent in writing was obtained from all the study participants. Privacy and confidentiality of the study participants were maintained at all stages of the study.

## Results

### Sample characteristics

We surveyed 475 post-partum mothers from three districts in Tamil Nadu. Among the study sample more than 60% of the mothers delivered within six-months prior to the survey. Majority (65.5%) delivered in a public facility, more than half had a caesarean section, 44.8% (n = 213) were primigravida, and 55.2% (n = 262) were multigravida mothers (Table 1).

**Table 1:** Characteristics of the study sample (n = 475).

Study Variables	Frequency (N)	(%)
**District**		
Tuticorin	160	33.7
Kanyakumari	156	32.8
Pudukkottai	159	33.5
**Place of residence**		
Urban	240	50.5
Rural	235	49.5
**Religion**		
Christian	62	13.1
Hindu	380	80.0
Muslim	33	6.9
**Caste**		
General	49	10.3
OBC	299	62.9
SC/ST	127	26.7
**Employment Status**		
Not employed	444	93.5
Employed	31	6.5
**Wealth Index**		
Lower	198	41.7
Upper lower	137	28.8
Middle to upper	140	29.5
**Education**		
Up to high school	112	23.6
Intermediate or diploma	109	22.9
Graduate, professional or honours	254	53.5
**Gravida**		
Primigravida	213	44.8
Multigravida	262	55.2
**Place of ANC visits**		
Public sector	311	65.5
Private sector	164	34.5
High risk pregnancy*		
No	266	56.0
Yes	209	44.0
**Intrapartum complications** ^**#**^		
No	444	93.5
Yes	31	6.5
**Place of delivery**		
Public sector	314	66.1
Private sector	161	33.9
**Female Sterilisation**		
No	345	72.6
Yes	130	27.4
**Received financial assistance**		
No	151	31.8
Yes	324	68.2
**Out of pocket expenses**		
Up to INR 10000^#^	284	59.8
>INR10000	191	40.2
**Took loans or sold assets to pay OOPE**		
No	281	59.2
Yes	194	40.8
**Time since delivery**		
0-3 months	146	30.7
3.1 to 6 months	152	32.0
6.1 to 9 months	86	18.1
9.1 to 12 months	91	19.2

*includes severe anaemia, pre-eclampsia, Gestational Diabetes, Hypothyroidism, Young Primi or Elderly Gravida, Previous C-Section, History of systematic illness etc.; #includes breech presentation, prolonged labour, excessive bleeding; OBC = Other Backward Castes, SC = Scheduled Caste, ST = Scheduled Tribes; ANC = Antenatal Care; OOPE=Out of pocket expenses; INR = Indian Rupees; #approximately 120 United States Dollar as per the INR to USD conversion rate during the study period

### Prevalence and indications of C-section deliveries

The C-sections accounted for more than half of all deliveries (52.6%, 95%CI = 48.1–57.1). Among the mothers who had vaginal delivery (n = 225), 18.2% (95%CI = 13.6–23.6) had a high-risk pregnancy, whereas among the mothers who underwent a C-section (n = 250), 67.2% (95% CI = 60.3–72.0) had a high-risk pregnancy. Among these mothers, previous C-section delivery was a major indication of high-risk pregnancy (41.2%, 95%CI = 35.2–47.4), followed by preeclampsia (12.4%, 95%CI = 8.7–16.9) and hypothyroidism (7.6%, 95%CI = 4.7–11.3) (Table 2).

**Table 2:** Distribution of high-risk pregnancy indications and intrapartum complications by mode of delivery.

		Vaginal Delivery (n = 225)		C-Section Delivery (n = 250)			
Groups	Indications	n	Percentage (95%CI)	n Planned C-Section^a^	n Emergency C-Section^b^	n Total C-Sections (a + b)	Percentage (95%CI)
**Indications for High-Risk Pregnancy**	Severe Anaemia (HB < 7 g/dL)	7	3.1 (1.3–5.9)	12	0	12	4.8 (2.6–7.9)
	Preeclampsia	8	3.6 (1.6–6.5)	27	4	31	12.4 (8.7–16.9)
	Gestational DM	6	2.7 (1.1–5.3)	17	0	17	6.8 (4.1–10.4)
	Hypothyroidism	7	3.1 (1.3–5.9)	17	2	19	7.6 (4.7–11.3)
	Young Primigravida/Elderly gravida	4	1.8 (0.6–4.1)	7	1	8	3.2 (1.5–5.9)
	Twin/Multiple pregnancy	0	0	3	0	3	1.2 (0.3–3.1)
	Malpresentation	0	0	2	0	2	0.8 (0.1–2.4)
	Previous C-section delivery	1	0.4 (0.001–1.9)	102	1	103	41.2 (35.2–47.4)
	Placenta previa	0	0	6	0	6	2.4 (1.0–4.8)
	Previous bad obstetric history	0	0	6	0	6	2.4 (1.0–4.8)
	Rh negative	2	0.9 (0.1–2.7)	3	1	4	1.6 (0.5–3.7)
	Short stature	10	4.4 (2.3–7.7)	16	2	18	7.2 (4.4–10.8)
	Obesity	0	0 (0.0–0.0)	4	1	5	2 (0.7–4.2)
	History of systemic illness	6	2.7 (1.1–5.3)	11	0	11	4.4 (2.3–7.4)
**Intrapartum complications**	Breech Presentation	0	0	18	0	18	7.2 (4.4–10.8)
	Prolonged Labour	1	0.4 (0.01–1.9)	0	7	7	2.8 (1.2–5.3)
	Excessive Bleeding	3	1.3 (0.3–3.4)	2	0	2	0.8 (0.1–2.4)
**High risk pregnancy**	Yes	41	18.2 (13.6–23.6)	155	11	166	67.2 (60.3–72.0)
	No	184	81.8 (76.4–86.4)	81	3	84	32.8 (27.2–38.9)
	Total*	**225**		**236**	**14**	**250**	

C-section = Caesarean Section; a = planned C-section is defined as a C-section when doctor/expectant mother decided to undertake/undergo the C-section prior to the onset of labour, with or without an indication of high-risk pregnancy/intrapartum complication. b = emergency C-section was defined as a C-section when doctor decided to undertake an emergency C-section after the onset of labour; *mothers had more than one indications for high-risk pregnancy/intrapartum complications. HB = Haemoglobin, DM = Diabetes Mellitus, Rh = Rhesus factor; CI = Confidence Interval

### Factors associated with C-section deliveries

Mothers with a high-risk pregnancy had significantly higher odds (AOR = 17.21, 95% CI = 7.57–39.03, p < 0.001) of C-section deliveries. Similarly, mothers delivering in a private healthcare facility (AOR = 3.02, 95% CI = 1.62–5.65, p < 0.001), and primigravida mothers (AOR = 3.76, 95% CI = 1.96–7.20, p < 0.001) have higher odds of C-section deliveries (Table 3). The logistic regression had an acceptable model fit (Hosmer-Lemeshow test, p = 0.712).

**Table 3:** Factors associated with caesarean section deliveries (n = 475).

	Type of delivery		AOR (95% CI)	p-value
	Vaginal delivery (225) %	Caesarean section (250) %		
**District**				
Pudukkottai (ref)	49.7	50.3		
Tuticorin	56.3	43.8	0.61 (0.39–0.94)	0.024
Kanyakumari	35.9	64.1	1.69 (1.06–2.69)	0.028
**Place of residence**				
Urban (ref)	46.3	53.8		
Rural	48.5	51.5	1.78 (1.23–2.58)	0.002
**High risk pregnancy**				
No (ref)	68.7	31.3		
Yes	19.8	80.2	17.21 (7.57–39.13)	<0.001
**Place of delivery**				
Public (ref)	56.4	43.6		
Private	29.8	70.2	3.02 (1.62–5.65)	0.001
**Gravida**				
Multigravida (ref)	47.4	52.6		
Primigravida	47.3	52.7	3.76 (1.96–7.20)	<0.001
**Female Sterilisation**				
No (ref)	52.2	47.8		
Yes	34.6	65.4	1.81 (1.20–2.73)	0.005

Hosmer and Lemeshow test, p = 0.7124. Mean VIF = 1.49 Dependent variable: Mode of delivery: vaginal delivery (ref), caesarean section; Ref = Reference Category. The model had an AUROC of 0.8406, Sensitivity = 78.40%, Specificity = 75.11%, Positive Predictive Value = 77.78%, and Negative Predictive Value = 75.78%.

### Association of mode of delivery with household finances and HRQL

50.4% of mothers with C-section reported household experienced distress financing (i.e., taking loans or selling assets to pay OOPE), compared to 30.2% of mothers with vaginal delivery (χ^2^ = 19.955, df = 1, p < 0.001). Further, mother who underwent a C-section incurred higher OOPE (p < 0.001), and had poorer health related quality of life scores in PCS (p < 0.001) and MCS (p = 0.001) domains (Table 4).

**Table 4:** Financial implications and health related quality of life (HRQL) among vaginal and C-section deliveries: a comparison (n = 475).

Costs and HRQL variables	Vaginal delivery (n = 225)		C-Section (n = 250)		p-value*
	Mean (95% CI)	Median (IQR)	Mean	Median (IQR)	
OOPE Payments (in INR)	13814 (11189–16439)	5000 (10920)	39608 (31673–47543)	10550 (60180)	<0.001
Cost for post-natal care and treatment (in INR)	1377 (1262–2820)	200 (2000)	2041 (1262–2820)	250 (50000)	0.974
Total wage loss to the family care giver in INR (n = 157)	14887 (12181–17593)	12000 (13000)	19012 (14816–23209)	14500 (20313)	0.260
Amount received as financial assistance in INR (n = 324)	6909 (5799–8019)	4000 (4000)	6440 (5297–7584)	4000 (4000)	0.058
SF-12 PCS	49.54 (48.60–50.47)	51.46 (9.52)	42.39 (41.08–43.70)	44.38 (16.96)	<0.001
SF-12 MCS	55.33 (54.37–56.31)	58.79 (8.03)	53.12 (51.99–54.25)	55.93 (9.82)	0.001

*significance of difference between the groups was assessed using Mann-Whitney U test; INR = Indian Rupees, OOPE= Out of pocket expenses; SF 12-PCS = Short Form Health Survey-Physical Component Summary; SF 12-MCS = Short Form Health Survey-Mental Component Summary.

The multivariate binary logistic regression found a significant variation in OOPE (Hosmer-Lemeshow test, p = 0.386) for institutional deliveries across districts, with those residing in Kanyakumari having an AOR of 5.73 (95%CI = 4.07–8.06, p < 0.001) for incurring OOPE over INR.10000/- compared to the women who resided in Pudukkottai. While rural women were less likely to incur OOPE > INR.10000/- (AOR = 0.70, 95%CI = 0.56–0.87, p = 0.002), better socio-economic status in terms of graduate, professional or honours level education (AOR = 2.56, 95%CI = 1.38–4.74, p = 0.03) and wealth index of middle to upper income level (AOR = 4.75, 95%CI = 2.27–9.94, p < 0.001) were associated with OOPE above INR.10000/-. Women who underwent a C-section (AOR = 2.05, 95% CI = 1.22–3.47, p = 0.007) had a greater probability to incur OOPE over INR.10000/- compared to women who undergo vaginal delivery (Table 5).

**Table 5:** Factors associated with OOPE expenses: the role of mode of delivery (n = 475).

Independent Variables	OOPE Mean (95% CI)	Median OOPE (INR)	OOPE Range (INR)		AOR (95% CI)	p-value
**District**			**Min**	**Max**		
Pudukkottai (ref)	23800 (18297–29302)	6000	1000	199000		
Tuticorin	17683 (13394–21972)	5005	0.00	160200	1.91 (1.30–2.79)	0.001
Kanyakumari	41004 (29518–52491)	33450	1900	826000	5.73 (4.07–8.06)	<0.001
**Place of residence**						
Urban (ref)	31796 (21043–36550)	8100	1000	271000		
Rural	22890 (15212–30568)	6800	0.00	826000	0.70 (0.56–0.87)	0.002
**Education**						
Up to high school (ref)	16643 (10392–22893)	5000	1200	271000		
Intermediate or diploma	18405 (12454–24356)	5000	200	199000	0.91 (0.32–2.63)	0.864
Graduate, professional or honours	35984 (28580–43388)	14875	0	826000	2.56 (1.38–4.74)	0.003
**Wealth Index**						
Lower (ref)	13057 (10171–15943)	5000	0	117000		
Upper lower	25861 (19112–32610)	71000	1000	271000	1.72 (0.87–3.40)	0.121
Middle to upper	49157 (36714–61560)	46550	2000	826000	4.75 (2.27–9.94)	<0.001
**Mode of delivery**						
Vaginal delivery (ref)	13814 (11189–16439)	5000	0	148000		
C-Section	39608 (31673–47543)	10550	0	826000	2.05 (1.22–3.47)	0.007

Hosmer and Lemeshow test, p = 0.3862; Mean VIF = 1.44; OOPE= Out of pocket expenditure, INR = Indian rupees, AOR = Adjusted odds ratio, CI = Confidence Interval. Dependent variable: Out of pocket expenditure: up to INR 10000 (ref), > INR 10000; Ref = Reference Category. AUROC = 0.8152; Sensitivity- 61.26%, Specificity-82.75%, Positive predictive value-70.48%, Negative predictive value-76.05%. **Note**: Binary logistic regression analysis was conducted to undertake multivariate analysis; place of delivery was excluded from the model as it had strong multicollinearity with C-Section. INR 10000 is equivalent to approximately 120 United States Dollar as per the INR to USD conversion rate during the study period.

With respect to health related quality of life, post-partum mothers belonging to middle to upper wealth index were had a lower odds of good HRQL in PCS component (AOR = 0.60, 95%CI = 0.49–0.74, p < 0.001), C-section was associated with poor HRQL in PCS component (AOR = 0.31, 95% CI = 0.18–0.55, p < 0.001). Also, greater time duration (9.1 to 12 months) since delivery is associated with good HRQL in PCS component (AOR = 4.24, 95%CI = 2.17–8.28, p < 0.001) [Hosmer-Lemeshow test, p = 0.368] (Table 6).

**Table 6:** Factors associated with Health-Related Quality of Life: the role of mode of delivery (n = 475).

	PCS		MCS	
	AOR (95%CI)	p-value	AOR (95%CI)	p-value
**Education**				
Up to high school (ref)				
Intermediate or diploma	2.15 (1.38–3.35)	0.001	1.76 (0.63–4.92)	0.278
Graduate, professional or honours	1.24 (0.64–2.40)	0.534	1.08 (0.69–1.40)	0.735
**Wealth Index**				
Lower (ref)				
Upper lower	0.69 (0.51–0.94)	0.017	0.87 (0.68–1.11)	0.278
Middle to upper	0.67 (0.52–0.86)	0.002	0.96 (0.65–1.40)	0.735
**Mode of delivery**				
Vaginal delivery (ref)				
C-Section	0.31 (0.18–0.55)	<0.001	0.68 (0.33–1.38)	0.283
**Time since delivery**				
0-3 months (ref)				
3.1 to 6 months	1.59 (1.19–2.12)	0.002	0.75 (0.41–1.37)	0.351
6.1 to 9 months	4.07 (2.18–7.59)	<0.001	1.30 (0.69–2.44)	0.417
9.1 to 12 months	4.24 (2.17–8.28)	<0.001	1.15 (0.53–2.51)	0.722

Hosmer and Lemeshow test, (PCS: p = 0.3685; MCS: p = 0.3709); Mean VIF: PCS = 1.43; MCS = 1.43; AOR = Adjusted odds ratio, CI = Confidence Interval; Dependent variable: PCS: poor (ref), good; MCS: poor (ref), good; ref = Reference Category; PCS = “Physical component Summary” of SF-12 measuring physical functioning, bodily pain, and general health; MCS = “Mental component summary” of SF-12 measuring vitality, social functioning, role-emotional and mental health.

PCS: AUROC = 0.7440; sensitivity: 56.54%, specificity= 81.61%, positive predictive value = 71.60%, negative predictive value= 69.61%.

MCS: AUROC = 0.6317; sensitivity= 100%, specificity= 0.00%, positive predictive value= 80.21%, Negative predictive value=.%.

## Discussion

The observed prevalence of C-section deliveries (52.6%) in our study was higher than the 44.9% for Tamil Nadu reported in NFHS-5 [5]. The difference could be attributed to the sampled districts which are known to have higher C-section prevalence [16,28,29]. The WHO statement on C-sections indicate that the C-section rates above 10% at the population level does not substantially decrease MMR [8]. Systematic reviews suggest that the optimal C-section rates across the countries should be between 9–19% [30]. However, the C-section rates in Indian states, especially Tamil Nadu are rapidly rising [4,5]. Improved economic status, private healthcare use, reducing fertility rates, and medicalisation are known to be associated with high C-section rates [31], most of which can be observed in case of Tamil Nadu.

Factors such as high-risk pregnancy, place of delivery, and being a primigravida were associated with C-section deliveries. While high risk pregnancies are considered an indication for C-section, research based on nationally representative surveys report that close to half of all pregnancies in India can be categorised as high-risk [9]. In regions with high coverage of ANC and institutional deliveries, this high prevalence of high-risk pregnancies could be translating to increasing C-section deliveries. A major contributor to high-risk pregnancies is the previous history of C-section, suggesting that the C-sections among Primigravida mothers will turn out to be a major contributor for C-section in subsequent deliveries. Further, beyond prior C-section, other key indicators for high-risk pregnancies including short birth-spacing, moderate to severe anaemia, obesity and comorbidities also continue to be associated with C-section deliveries (S1 File, Table 4) and are preventable through targeted public health interventions [9]. Similar to earlier studies, our study also found that primigravida mothers have a greater odds of C-section deliveries [32,33].

Place of delivery was also associated with the mode of delivery, with mothers delivering at a private healthcare facility having a higher odds of undergoing a C-section compared to those delivering at a public facility [34,35]. From 1992 to 2016 caesarean section deliveries in private facilities in India increased by 232.52% (12.30% in 1992 to 40.9% in 2016), while those at public facilities increased by 65.28% (7.20% in 1992 to 11.90% in 2016) [36]. While our study did not explore the reasons for high C-section rates in private hospitals, evidence from other south Indian states reported the high prevalence of C-section deliveries in private settings attributable to practice of defensive medicine, referral of high-risk pregnancies to private hospitals, and the preference for C-sections [37]. Despite a higher proportion in urban areas, we observed that mothers from rural regions had higher odds of undergoing C-section compared to their urban counter parts. This finding contradicts other studies which reported that urban regions have a greater proportion of women who underwent C-section [38]. The difference observed in our study could be specific to the context of our study, statistical interaction between covariates, and similar settings like Tamil Nadu and other south Indian states wherein a higher proportion of C-sections in rural areas were reported [39]. Further studies are required to investigate this difference.

The average OOPE of C-section was found to be INR 39608/- more than twice the cost of vaginal delivery, with 50.4% of the participants undergoing C-section having to pay more than INR 10000/- rupees. Our estimate exceeded the average cost of C-section deliveries reported in India’s NFHS-5, which was INR 7,304 in public facilities and INR 36,595 in private facilities [7]. Similar to earlier research, our findings also indicate that half of the participants who delivered through C-section underwent distress financing by either taking loans or selling assets to pay OOPE, while 30% of the participants undergoing vaginal delivery faced such financial implications [7]. While we did not study the OOPE attributed to C-section as part of the total health expenditures or household expenditure, a 2019 study based on NFHS concluded that C-section deliveries have a high predictive probability of distress financing with the states of Telangana and Tamil Nadu with high percentage of households selling assets or borrowing to pay for institutional delivery [19]. Similar findings were made in other developing country settings where catastrophic health expenses associated with C-sections ranged from 27% to 77% [40].

With respect to Health-Related Quality of Life, C-section deliveries are associated with lower HRQL, a finding supported by other studies. Similarly, time since delivery was associated with PCS component of HRQL. A 2022 systematic review and meta-analysis of 21 studies reported that compared to C-sections, women undergoing vaginal deliveries had better HRQL in the components of physical role, physical functioning and social functioning [27]. Post-natal mothers who had vaginal births including those with episiotomy, and assisted vaginal delivery had higher physical and mental QOL compared to mothers with C-section deliveries [41,42]. C-section has been associated with chronic pelvic pain, longer time for functional recovery, and post-surgical pain, and resultant psychosocial impact [41,43,44].

Altogether, our study has important policy implications for maternal health programmes in India, particularly in states like Tamil Nadu. While C-sections remain a critical life-saving intervention when medically indicated, the high prevalence observed in this study coupled with significant OOPE and poor postpartum HRQL outcomes underscores the need for strengthening regulatory and programmatic responses. Policy efforts should focus on improving adherence to evidence-based clinical guidelines, strengthening audit and monitoring mechanisms for C-section deliveries in healthcare facilities, with special focus on private institutions. Preventive public health strategies aimed at reducing avoidable high-risk pregnancies (such as improving maternal nutrition, addressing anaemia, encouraging optimal birth spacing etc.) can help to reduce unwarranted C-sections. Expanding the financial protection mechanisms like Pradhan Mantri Jan Arogya Yojana to comprehensively cover child birth can facilitate to mitigate financial distress associated with C-sections.

This study has several limitations. First, the cross-sectional design prevents establishing causal relationships between the exposures and outcomes examined. Second, information on out-of-pocket expenditures and other key variables was based on self-report and may be subject to recall bias. Similarly, limited studies on validity of SF-12 cut-off among post-partum mothers, and the potential for responses to the SF-12 quality-of-life instrument to have been influenced by social desirability bias are limitations. Although we adjusted for a range of relevant covariates in our regression models, residual confounding from unmeasured or inadequately measured variables including cultural factors, physicians’ preference for C-section, fear of litigation etc. cannot be ruled out. Also, the study clusters were drawn from three districts of Tamil Nadu, which may limit the generalizability of the findings to other regions with different health system characteristics, socioeconomic contexts, or patterns of healthcare utilization. The sample size calculation did not account for a design effect despite the use of a multistage cluster sampling approach. As a result, the required sample size may have been underestimated, which could affect the precision of the estimates.

## Conclusions

The high prevalence of C-section deliveries observed in this study underscores the importance of closely monitoring obstetric practices, particularly in private healthcare settings. High-risk pregnancies and private facility deliveries were associated with greater probability of C-section delivery. Furthermore, C-sections were associated with higher out-of-pocket expenditures and poorer physical health-related quality of life among post-partum mothers. Future research is required to examine the role of public health policies in optimizing C-section deliveries. Additionally, the market dynamics of C-section deliveries in context of prevalent high-risk pregnancies and private sector participation in Indian healthcare ecosystem needs further investigation.

## Supporting information

S1 File Supplementary tables. (DOCX)

S2 File Dataset. (XLS)
