# Supplementary material for: Caesarean deliveries and their impact on financial burden and quality of life among post-partum mothers in Tamil Nadu, India
Source: PLoS One. 2026 Sep 22;21(9):e0358778. doi: 10.1371/journal.pone.0358778 (PMC13596793; doi:10.1371/journal.pone.0358778)
Supplement: Supplementary file 1 [file pone.0358778.s003.pdf]

This is a well-conceived and relevant study that addresses a timely and important issue in maternal health in India—the growing prevalence of Caesarean section (C-section) deliveries and their financial and quality-of-life implications. The topic is significant from both public health and health economics perspectives, and the paper provides useful evidence from a state known for its high institutional delivery rates. However, there are several methodological, analytical, and interpretive concerns that need to be addressed before the paper can be considered for publication.

Major Comments

Study Rationale and Novelty

- The introduction establishes context but needs a stronger justification of the study's **novel contribution**. Similar studies have been conducted in Indian states using NFHS data. The authors should clarify how their primary data collection adds value (e.g., inclusion of HRQL outcomes, district-level insights, or cost breakdowns).
- Please clarify how this study adds to prior work by Singh et al. (2023, *BMC Health Services Research*) and Mishra & Mohanty (2019, *Int J Equity Health*), both of which examined OOPE for C-sections in India.

Study Design and Sampling

- The **sampling strategy** needs clearer explanation. Although a multistage stratified cluster random sampling is described, the rationale for selecting only three districts may introduce selection bias. Please justify how these districts represent Tamil Nadu overall.
- Clarify whether the sampling was proportional to population size.
- The **sample size calculation** should explicitly mention the formula used and parameters (confidence interval, expected prevalence, precision, non-response rate).

Measurement and Data Collection

- The use of self-reported data for C-section and OOPE introduces potential recall bias, particularly among mothers who delivered up to a year prior. Discuss this limitation.
- The SF-12 tool is appropriate but please specify scoring and cut-off criteria (e.g., how PCS and MCS were standardized to a mean of 50). Reference to the validated Tamil version, if available, would strengthen credibility.

Statistical Analysis

- The choice of logistic regression is sound; however, the inclusion/exclusion of variables needs clearer justification. Were collinearity diagnostics performed?
- OOPE was dichotomized using the median, but a sensitivity analysis (e.g., log-transformed continuous outcome) could provide additional robustness.
- In Table 5, the inclusion of district, education, and wealth index together with mode of delivery may cause multicollinearity; explain variance inflation factors or other checks.
- In table 2, it requires consistency for presenting CI, not like 0.4 (**0.00-1.9**)

- The authors should report model goodness-of-fit statistics (e.g., Hosmer-Lemeshow test) in the results narrative.

Results Presentation

- Tables are comprehensive but could be condensed. Consider moving descriptive details (e.g., high-risk pregnancy indicators) to supplementary material.
- It would strengthen the paper to present mean OOPE by delivery mode and effect sizes (mean differences with confidence intervals), not only odds ratios.

Interpretation and Discussion

- The discussion appropriately references prior literature, but the policy implications section could be expanded. For example, discuss how insurance schemes (like PM-JAY) or maternal benefit programs might mitigate financial distress.
- Some statements imply causality ("C-section reduces HRQL") despite cross-sectional data — please rephrase these as **associations**, not effects.
- Include potential confounders such as type of facility (public/private), maternal complications, or socioeconomic status in the causal discussion.

Data Availability and Ethical Statement

- The current data availability statement ("available upon reasonable request") does not fully comply with *PLOS ONE*'s open data policy. The dataset (de-identified) should be deposited in a public repository if possible.

Minor Comments

Abstract:

- Please report the study period (April–June 2023) in the Methods section of the abstract.
- The abstract is concise but contains typographical errors ("finical" → "financial").

Writing and Formatting

- Some typographical and grammatical inconsistencies (e.g., spacing after periods, capitalization of "C-section") need minor proofreading.
- Ensure consistent use of "C-section" versus "Caesarean section".

References:

- Ensure all references are formatted according to PLOS ONE guidelines.
- Some references (e.g., 4, 5, 6) appear to cite websites; ensure all are correctly formatted according to *PLOS ONE* style.
- References are comprehensive, though some older citations could be replaced with more recent evidence (2023–2024) where available.

Limitations

- The limitations section should be expanded to discuss: (i) potential reporting bias, (ii) residual confounding, and (iii) the restriction to only three districts.

Recommendation

Major Revision

The manuscript presents valuable findings with strong public health relevance but requires improvements in methodological clarity, statistical robustness, and cautious interpretation. Addressing the above concerns will substantially strengthen the paper's contribution to the evidence base on the economic and quality-of-life implications of C-section deliveries in India.
