# Supplementary material for: Caesarean deliveries and their impact on financial burden and quality of life among post-partum mothers in Tamil Nadu, India
Source: PLoS One. 2026 Sep 22;21(9):e0358778. doi: 10.1371/journal.pone.0358778 (PMC13596793; doi:10.1371/journal.pone.0358778)
Supplement: Supplementary file 2 [file pone.0358778.s001.docx]

**Logistic Regression Analysis-Full Models**

**Table 1**: Factors Associated with Caesarean section deliveries (n=475)

|  | **Type of delivery** | |  |  |
| --- | --- | --- | --- | --- |
|  | **Vaginal delivery (225)** | **Caesarean section (250)** | **AOR (95% CI)** | **p-value** |
| **District** | **%** | **%** |  |  |
| Pudukkottai (ref) | 49.7 | 50.3 |  |  |
| Tuticorin | 56.3 | 43.8 | 0.61 (0.39-0.94) | 0.024 |
| Kanyakumari | 35.9 | 64.1 | 1.69 (1.06-2.69) | 0.028 |
| **Place of residence** |  |  |  |  |
| Urban (ref) | 46.3 | 53.8 |  |  |
| Rural | 48.5 | 51.5 | 1.78 (1.23-2.58) | 0.002 |
| **Wealth Index** |  |  |  |  |
| Lower (ref) | 51.0 | 49.0 |  |  |
| Upper lower | 49.6 | 50.4 | 0.98 (0.54-1.89) | 0.975 |
| Middle to upper | 40.0 | 60.0 | 1.20 (0.65-2.21) | 0.558 |
| **Education** |  |  |  |  |
| Up to high school (ref) | 45.5 | 54.5 |  |  |
| Intermediate or diploma | 53.2 | 46.8 | 0.71 (0.36-1.38) | 0.314 |
| Graduate, professional or honours | 45.7 | 54.3 | 0.79 (0.37-1.72) | 0.557 |
| **High risk pregnancy** |  |  |  |  |
| No (ref) | 68.7 | 31.3 |  |  |
| Yes | 19.8 | 80.2 | 17.21 (7.57-39.13) | <0.001 |
| **Place of delivery** |  |  |  |  |
| Public (ref) | 56.4 | 43.6 |  |  |
| Private | 29.8 | 70.2 | 3.02 (1.62-5.65) | 0.001 |
| **Gravida** |  |  |  |  |
| Multigravida (ref) | 47.4 | 52.6 |  |  |
| Primigravida | 47.3 | 52.7 | 3.76 (1.96-7.20) | <0.001 |
| **Female Sterilisation** |  |  |  |  |
| No (ref) | 52.2 | 47.8 |  |  |
| Yes | 34.6 | 65.4 | 1.81 (1.20-2.73) | 0.005 |
| Hosmer and Lemeshow test, p=0.7124. Mean VIF= 1.49 Dependent variable: Mode of delivery: vaginal delivery (ref), caesarean section; Ref=Reference Category. The model had an AUROC of 0.8406, Sensitivity=78.40%, Specificity=75.11%, Positive Predictive Value=77.78%, and Negative Predictive Value=75.78%. | | | | |

**Figure 1:** Area under ROC curve for factors associated with C-section deliveries

**Table 2:** Factors associated with OOPE expenses: the role of mode of delivery (n=475)

| **Independent Variables** | **Median OOPE (INR)** | **OOPE Range (INR)** | | **AOR (95% CI)** | **p-value** |
| --- | --- | --- | --- | --- | --- |
| **District** |  | **Min** | **Max** |  |  |
|  |  |  |  |  |  |
| Pudukkottai (ref) | 6000 | 1000 | 199000 |  |  |
| Tuticorin | 5005 | 0.00 | 160200 | 1.91 (1.30-2.79) | 0.001 |
| Kanyakumari | 33450 | 1900 | 826000 | 5.73 (4.07-8.06) | <0.001 |
| **Place of residence** |  |  |  |  |  |
| Urban (ref) | 8100 | 1000 | 271000 |  |  |
| Rural | 6800 | 0.00 | 826000 | 0.70 (0.56-0.87) | 0.002 |
| **Education** |  |  |  |  |  |
| Up to high school (ref) | 5000 | 1200 | 271000 |  |  |
| Intermediate or diploma | 5000 | 200 | 199000 | 0.91 (0.32-2.63) | 0.864 |
| Graduate, professional or honours | 14875 | 0 | 826000 | 2.56 (1.38-4.74) | 0.003 |
| **Wealth Index** |  |  |  |  |  |
| Lower (ref) | 5000 | 0 | 117000 |  |  |
| Upper lower | 71000 | 1000 | 271000 | 1.72 (0.87-3.40) | 0.121 |
| Middle to upper | 46550 | 2000 | 826000 | 4.75 (2.27-9.94) | <0.001 |
| **High risk pregnancy** |  |  |  |  |  |
| No (ref) | 8000 | 1000 | 350000 |  |  |
| Yes | 10000 | 2000 | 850000 | 1.45 (0.86-2.44) | 0.163 |
| **Gravida** |  |  |  |  |  |
| Primigravida(ref) | 7000 | 0 | 826000 |  |  |
| Multigravida | 7000 | 0 | 271000 | 1.03 (0.75-1.40) | 0.861 |
| **Experienced Intrapartum complications** |  |  |  |  |  |
| No (ref) | 7000 | 0.00 | 826000 |  |  |
| Yes | 6000 | 0.00 | 87900 | 0.54 (0.21-1.36) | 0.192 |
| **Mode of delivery** |  |  |  |  |  |
| Vaginal delivery (ref) | 5000 | 0 | 148000 |  |  |
| C-Section | 10550 | 0 | 826000 | 2.05 (1.22-3.47) | 0.007 |
| Hosmer and Lemeshow test, p=0.3862; Mean VIF=1.44; OOPE= Out of pocket expenditure, INR= Indian rupees, AOR=Adjusted odds ratio, CI= Confidence Interval. Dependent variable: Out of pocket expenditure: up to INR 10000 (ref), > INR 10000; Ref=Reference Category. AUROC= 0.8152; Sensitivity- 61.26%, Specificity-82.75%, Positive predictive value-70.48%, Negative predictive value-76.05%. Note: Binary logistic regression analysis was conducted to undertake multivariate analysis; place of delivery was excluded from the model as it had strong multicollinearity with C-Section. INR 10000 is equivalent to approximately 120 United States Dollar as per the INR to USD conversion rate during the study period; | | | | | |

**Figure 2**: Area under ROC curve for factors associated with OOPE expenditures

**Table 3:** Factors associated with Health-Related Quality of Life: the role of mode of delivery (n=475)

|  | **PCS** | | **MCS** | |
| --- | --- | --- | --- | --- |
|  | **AOR (95%CI)** | **p-value** | **AOR (95%CI)** | **p-value** |
| **Place of residence** |  |  |  |  |
| Urban (ref) |  |  |  |  |
| Rural | 1.12 (0.55-2.25) | 0.759 | 1.45 (0.90-2.31) | 0.124 |
| **Education** |  |  |  |  |
| Up to high school (ref) |  |  |  |  |
| Intermediate or diploma | 2.15 (1.38-3.35) | 0.001 | 1.76 (0.63-4.92) | 0.278 |
| Graduate, professional or honours | 1.24 (0.64-2.40) | 0.534 | 1.08 (0.69-1.40) | 0.735 |
| **Wealth Index** |  |  |  |  |
| Lower (ref) |  |  |  |  |
| Upper lower | 0.69 (0.51-0.94) | 0.017 | 0.87 (0.68-1.11) | 0.278 |
| Middle to upper | 0.67 (0.52-0.86) | 0.002 | 0.96 (0.65-1.40) | 0.735 |
| **Gravida** |  |  |  |  |
| Primigravida(ref) |  |  |  |  |
| Multigravida | 0.88 (0.57-1.36) | 0.571 | 1.11 (0.71-1.74) | 0.644 |
| **High risk pregnancy** |  |  |  |  |
| No (ref) |  |  |  |  |
| Yes | 0.97 (0.64-1.49) | 0.898 | 0.77 (0.43-1.40) | 0.401 |
| **Mode of delivery** |  |  |  |  |
| Vaginal delivery (ref) |  |  |  |  |
| C-Section | 0.31 (0.18-0.55) | <0.001 | 0.68 (0.33-1.38) | 0.283 |
| **Time since delivery** |  |  |  |  |
| 0-3 months (ref) |  |  |  |  |
| 3.1 to 6 months | 1.59 (1.19-2.12) | 0.002 | 0.75 (0.41-1.37) | 0.351 |
| 6.1 to 9 months | 4.07 (2.18-7.59) | <0.001 | 1.30 (0.69-2.44) | 0.417 |
| 9.1 to 12 months | 4.24 (2.17-8.28) | <0.001 | 1.15 (0.53-2.51) | 0.722 |
| Hosmer and Lemeshow test, (PCS: p=0.3685; MCS: p=0.3709); Mean VIF: PCS=1.43; MCS=1.43; AOR=Adjusted odds ratio, CI= Confidence Interval; Dependent variable: PCS: poor (ref), good; MCS: poor (ref), good; ref=Reference Category; PCS= “Physical component Summary” of SF-12 measuring physical functioning, bodily pain, and general health; MCS= “Mental component summary” of SF-12 measuring vitality, social functioning, role-emotional and mental health.  PCS: AUROC = 0.7440; sensitivity: 56.54%, specificity= 81.61%, positive predictive value = 71.60%, negative predictive value= 69.61%.  MCS: AUROC = 0.6317; sensitivity= 100%, specificity= 0.00%, positive predictive value= 80.21%, Negative predictive value= .%. | | | | |

**Figure 3:** Area under ROC curve for factors associated with PCS and MCS components of HRQL.

**Sensitivity analysis conducted with exclusion of participants with previous history of C-section**

**Table 4**: Factors Associated with Caesarean section deliveries (n=371)

|  | **AOR (95% CI)** | **p-value** |
| --- | --- | --- |
| **District** |  |  |
| Pudukkottai (ref) |  |  |
| Tuticorin | 0.60 (0.39-0.94) | 0.024 |
| Kanyakumari | 1.90 (1.30-2.79) | 0.001 |
| **Place of residence** |  |  |
| Urban (ref) |  |  |
| Rural | 1.83 (1.29-2.61) | 0.001 |
| **Wealth Index** |  |  |
| Lower (ref) |  |  |
| Upper lower | 1.03 (0.56-1.89) | 0.918 |
| Middle to upper | 1.24 (0.70-2.21) | 0.459 |
| **Education** |  |  |
| Up to high school (ref) |  |  |
| Intermediate or diploma | 0.66 (0.25-1.70) | 0.394 |
| Graduate, professional or honours | 0.82 (0.28-2.44) | 0.734 |
| **High risk pregnancy** |  |  |
| No (ref) |  |  |
| Yes | 6.31 (3.28-12.12) | <0.001 |
| **Place of delivery** |  |  |
| Public (ref) |  |  |
| Private | 2.87 (1.16-7.11) | 0.023 |
| **Gravida** |  |  |
| Multigravida (ref) |  |  |
| Primigravida | 4.90 (2.49-9.65) | <0.001 |
| **Female Sterilisation** |  |  |
| No (ref) |  |  |
| Yes | 0.65 (1.27-1.55) | 0.333 |

**Table 5:** Factors associated with OOPE expenses: the role of mode of delivery (n=371)

| **Independent Variables** | **AOR (95% CI)** | **p-value** |
| --- | --- | --- |
| **District** |  |  |
|  |  |  |
| Pudukkottai (ref) |  |  |
| Tuticorin | 1.98 (1.49-2.62) | <0.001 |
| Kanyakumari | 6.12 (5.52-6.78) | <0.001 |
| **Place of residence** |  |  |
| Urban (ref) |  |  |
| Rural | 0.61 (0.55-0.67) | <0.001 |
| **Education** |  |  |
| Up to high school (ref) |  |  |
| Intermediate or diploma | 0.79 (0.23-2.71) | 0.702 |
| Graduate, professional or honours | 3.13 (1.48-6.60) | 0.003 |
| **Wealth Index** |  |  |
| Lower (ref) |  |  |
| Upper lower | 1.44 (0.73-2.84) | 0.294 |
| Middle to upper | 3.47 (1.55-7.79) | 0.003 |
| **High risk pregnancy** |  |  |
| No (ref) |  |  |
| Yes | 1.56 (0.84-2.91) | 0.159 |
| **Gravida** |  |  |
| Multigravida (ref) |  |  |
| Primigravida | 0.81 (0.51-1.29) | 0.375 |
| **Experienced Intrapartum complications** |  |  |
| No (ref) |  |  |
| Yes | 0.49 (0.17-1.43) | 0.194 |
| **Mode of delivery** |  |  |
| Vaginal delivery (ref) |  |  |
| C-Section | 2.51 (1.20-5.26) | 0.014 |

**Table 6:** Factors associated with out-of-pocket expenditure incurred for institutional delivery (n=475)

|  | **Out of Pocket Expenditure**  **Mean (95% CI)** | **β* Coefficient (95%CI)** | **p-value** |
| --- | --- | --- | --- |
| **District** |  |  |  |
| Pudukkottai (ref) | 23800 (18297-29302) |  |  |
| Tuticorin | 17683 (13394-21972) | 0.63 (0.58-0.67) | 0.000 |
| Kanyakumari | 41004 (29518-52491) | 0.07 (0.01-0.13) | 0.031 |
| **Place of residence** |  |  |  |
| Urban (ref) | 31796 (21043-36550) |  |  |
| Rural | 22890 (15212-30568) | -0.09 (-0.18-0.004) | 0.058 |
| **Education** |  |  |  |
| Up to high school (ref) | 16643 (10392-22893) |  |  |
| Intermediate or diploma | 18405 (12454-24356) | -0.01 (-0.41-0.40) | 0.965 |
| Graduate, professional or honors | 35984 (28580-43388) | 0.41 (-0.04-0.87) | 0.067 |
| **Wealth Index** |  |  |  |
| Lower (ref) | 13057 (10171-15943) |  |  |
| Upper lower | 25861 (19112-32610) | 0.33 (-0.09-0.75) | 0.097 |
| Middle to upper | 49157 (36714-61560) | 0.92 (0.65-1.20) | <0.001 |
| **High risk pregnancy** |  |  |  |
| No (ref) | 20736 (17463-24010) |  |  |
| Yes | 35858 (26608-45107) | 0.20 (-0.07-0.48) | 0.120 |
| **Gravida** |  |  |  |
| Primigravida (ref) | 30137 (21473-38780) |  |  |
| Multigravida | 25157 (21009-29305) | -0.07 (-0.29-0.15) | 0.437 |
| **Experienced Intrapartum complications** |  |  |  |
| No (ref) | 27451 (22708-32195) |  |  |
| Yes | 26507 (14380-38634) | -0.14 (-0.72-0.43) | 0.548 |
| **Mode of delivery** |  |  |  |
| Vaginal delivery (ref) | 13814 (11189-16439) |  |  |
| C-Section | 39608 (31673-47543) | 0.65 (0.39-0.90) | 0.001 |

*The outcome variable out of pocket expenditure captured in Indian rupees was log transformed to undertake linear regression.

**Table 7:** Factors associated with Health-Related Quality of Life: the role of mode of delivery (n=371)

|  | **PCS** | | **MCS** | |
| --- | --- | --- | --- | --- |
|  | **AOR (95%CI)** | **p-value** | **AOR (95%CI)** | **p-value** |
| **Place of residence** |  |  |  |  |
| Urban (ref) |  |  |  |  |
| Rural | 1.01 (0.50-2.06) | 0.972 | 1.63 (0.77-3.46) | 0.199 |
| **Education** |  |  |  |  |
| Up to high school (ref) |  |  |  |  |
| Intermediate or diploma | 2.17 (1.65-2.84) | <0.001 | 1.47 (0.35-6.15) | 0.595 |
| Graduate, professional or honours | 1.22 (0.72-2.05) | 0.459 | 1.02 (0.45-2.32) | 0.954 |
| **Wealth Index** |  |  |  |  |
| Lower (ref) |  |  |  |  |
| Upper lower | 0.77 (0.48-1.24) | 0.283 | 0.74 (0.45-1.19) | 0.213 |
| Middle to upper | 0.66 (0.40-1.11) | 0.120 | 0.81 (0.58-1.12) | 0.202 |
| **Gravida** |  |  |  |  |
| Multigravida (ref) |  |  |  |  |
| Primigravida | 0.94 (0.61-1.44) | 0.771 | 1.12 (0.60-2.11) | 0.716 |
| **High risk pregnancy** |  |  |  |  |
| No (ref) |  |  |  |  |
| Yes | 1.10 (0.66-1.84) | 0.701 | 0.70 (0.41-1.20) | 0.193 |
| **Mode of delivery** |  |  |  |  |
| Vaginal delivery (ref) |  |  |  |  |
| C-Section | 0.37 (0.21-0.65) | <0.001 | 0.59 (0.30-1.16) | 0.129 |
| **Time since delivery** |  |  |  |  |
| 0-3 months (ref) |  |  |  |  |
| 3.1 to 6 months | 1.52 (0.89-2.59) | 0.127 | 0.73 (0.37-1.46) | 0.377 |
| 6.1 to 9 months | 3.59 (2.14-6.04) | <0.001 | 1.57 (0.53-4.67) | 0.417 |
| 9.1 to 12 months | 4.80 (2.50-9.21) | <0.001 | 1.51 (0.55-4.22) | 0.424 |

**Comparison of Financial implications and health related quality of life (HRQL) in deliveries occurring in public and private hospitals.**

**Table 8:** Financial implications and health related quality of life (HRQL) of participants who delivered in public and private hospitals: a comparison (n=475)

|  | **Public (n=314)** | | **Private (n=161)** | | **p-value*** |
| --- | --- | --- | --- | --- | --- |
| **Costs and HRQL variables** | **Mean** | **Median** | **Mean** | **Median** |  |
| OOPE Payments (in INR) | 5888 | 4900 | 69325 | 58000 | <0.001 |
| Cost for post-natal care and treatment (in INR) | 1027 | 200 | 3092 | 400 | 0.019 |
| Total wage loss for the family care giver in INR (n=157) | 15451 | 12655 | 24448 | 14000 | 0.270 |
| Amount received as financial assistance in INR (n=324) | 6531 | 4700 | 7047 | 4000 | <0.001 |
| SF-12 PCS | 46.97 | 50.39 | 43.45 | 45.26 | 0.132 |
| SF-12 MCS | 54.56 | 57.89 | 53.41 | 56.66 | 0.043 |

*significance of difference between the groups was assessed using Mann-Whitney U test; INR=Indian Rupees, OOPE= Out of pocket expenses; SF 12-PCS= Short Form Health Survey-Physical Component Summary; SF 12-MCS= Short Form Health Survey-Mental Component Summary; **Note:** The estimates of expenses were rounded to nearest whole number.
