# Supplementary material for: Caesarean deliveries and their impact on financial burden and quality of life among post-partum mothers in Tamil Nadu, India
Source: PLoS One. 2026 Sep 22;21(9):e0358778. doi: 10.1371/journal.pone.0358778 (PMC13596793; doi:10.1371/journal.pone.0358778)
Supplement: Supplementary file 4 [file pone.0358778.s006.docx]

**Point by point response to review comments**

Respected Editor and Reviewers,

Thank you for the careful and thoroughly reviewing our manuscript. We sincerely appreciate the insightful comments and suggestions provided through the review process. We have carefully considered each comment and have made revisions as suggested.

We hereby submit a detailed point-by-point response to the reviewers’ comments for your kind consideration. The reviewer comments have been highlighted, and our responses are provided alongside them, with corresponding line numbers indicating where revisions have been made in the manuscript.

Sincerely

Corresponding Author

6. Review Comments to the Author

Please use the space provided to explain your answers to the questions above. You may also include additional comments for the author, including concerns about dual publication, research ethics, or publication ethics. (Please upload your review as an attachment if it exceeds 20,000 characters)

Reviewer #2: Thank you to the authors for thoroughly addressing my previous comments and improving the manuscript considerably. The revised version demonstrates improved methodological clarity, stronger statistical reporting, more cautious interpretation of findings, and a more comprehensive discussion of limitations. I also appreciate the detailed point-by-point responses and the additional analyses conducted in response to the reviewers’ suggestions.

Only a few minor editorial revisions remain. In particular, some residual causal language is still present in the abstract conclusion: “C-section deliveries had a substantial financial impact on the households and reduced the quality of life of post-partum mothers.” Given the cross-sectional nature of the study, this wording may imply causality. It would be more appropriate to revise this as: “C-section deliveries were associated with higher financial burden and poorer quality of life among post-partum mothers.”

Subject to these minor revisions, I recommend acceptance of the manuscript.

**Author response:** Respected reviewer, thank you for the suggestion. We agree with the suggestion. We have revised our abstract conclusion as per the revision suggested. The following revision was made to **lines 47 and 48** of abstract “*C-section deliveries and were associated with higher financial burden and poorer quality of life among post-partum mothers*.”
