# Supplementary material for: Caesarean deliveries and their impact on financial burden and quality of life among post-partum mothers in Tamil Nadu, India
Source: PLoS One. 2026 Sep 22;21(9):e0358778. doi: 10.1371/journal.pone.0358778 (PMC13596793; doi:10.1371/journal.pone.0358778)
Supplement: Peer review history

# Peer review: Decision Letter 0

**DOI:** 10.1371/journal.pone.0358778.r001

**Authors:** Denny John

25 Jan 2026

Caesarean Deliveries and their impact on financial burden and quality of life among post-partum mothers in Tamil Nadu, India

Dear Dr. Kodali,

Thank you for submitting your manuscript to PLOS ONE. After careful consideration, we feel that it has merit but does not fully meet PLOS ONE’s publication criteria as it currently stands. Therefore, we invite you to submit a revised version of the manuscript that addresses the points raised during the review process.

Please submit your revised manuscript by Mar 09 2026 11:59PM. If you will need more time than this to complete your revisions, please reply to this message or contact the journal office at plosone@plos.org. When you're ready to submit your revision, log on to https://www.editorialmanager.com/pone/ and select the 'Submissions Needing Revision' folder to locate your manuscript file.

- A letter that responds to each point raised by the academic editor and reviewer(s). You should upload this letter as a separate file labeled 'Response to Reviewers'.
- A marked-up copy of your manuscript that highlights changes made to the original version. You should upload this as a separate file labeled 'Revised Manuscript with Track Changes'.
- An unmarked version of your revised paper without tracked changes. You should upload this as a separate file labeled 'Manuscript'.

If you would like to make changes to your financial disclosure, please include your updated statement in your cover letter. Guidelines for resubmitting your figure files are available below the reviewer comments at the end of this letter.

If applicable, we recommend that you deposit your laboratory protocols in protocols.io to enhance the reproducibility of your results. Protocols.io assigns your protocol its own identifier (DOI) so that it can be cited independently in the future. For instructions see: https://journals.plos.org/plosone/s/submission-guidelines#loc-laboratory-protocols. Additionally, PLOS ONE offers an option for publishing peer-reviewed Lab Protocol articles, which describe protocols hosted on protocols.io. Read more information on sharing protocols at https://plos.org/protocols?utm_medium=editorial-email&utm_source=authorletters&utm_campaign=protocols.

We look forward to receiving your revised manuscript.

Kind regards,

Denny John

Academic Editor

PLOS One

Journal Requirements:

When submitting your revision, we need you to address these additional requirements.

1.Please ensure that your manuscript meets PLOS ONE's style requirements, including those for file naming. The PLOS ONE style templates can be found at

https://journals.plos.org/plosone/s/file?id=wjVg/PLOSOne_formatting_sample_main_body.pdf and

https://journals.plos.org/plosone/s/file?id=ba62/PLOSOne_formatting_sample_title_authors_affiliations.pdf

2. PLOS requires an ORCID iD for the corresponding author in Editorial Manager on papers submitted after December 6th, 2016. Please ensure that you have an ORCID iD and that it is validated in Editorial Manager. To do this, go to ‘Update my Information’ (in the upper left-hand corner of the main menu), and click on the Fetch/Validate link next to the ORCID field. This will take you to the ORCID site and allow you to create a new iD or authenticate a pre-existing iD in Editorial Manager.

3. In the online submission form, you indicated that “The study data will be made available to individual researchers/institutions upon a reasonable request to the corresponding author.”

All PLOS journals now require all data underlying the findings described in their manuscript to be freely available to other researchers, either 1. In a public repository, 2. Within the manuscript itself, or 3. Uploaded as supplementary information.

This policy applies to all data except where public deposition would breach compliance with the protocol approved by your research ethics board. If your data cannot be made publicly available for ethical or legal reasons (e.g., public availability would compromise patient privacy), please explain your reasons on resubmission and your exemption request will be escalated for approval.

4. If the reviewer comments include a recommendation to cite specific previously published works, please review and evaluate these publications to determine whether they are relevant and should be cited. There is no requirement to cite these works unless the editor has indicated otherwise.

**Note from the Editorial office:**  Upon internal evaluation of the reviews provided, we kindly request you to disregard the reviewer report provided by Reviewer 1. No amendments are required in response to reviewer 1’s comments’

[Note: HTML markup is below. Please do not edit.]

Reviewer's Responses to Questions

**Comments to the Author**

1. Is the manuscript technically sound, and do the data support the conclusions?

Reviewer #1: Yes

Reviewer #2: Yes

Reviewer #3: Partly

Reviewer #4: Partly

Reviewer #5: Yes

**********

2. Has the statistical analysis been performed appropriately and rigorously?

Reviewer #1: Yes

Reviewer #2: Yes

Reviewer #3: Yes

Reviewer #4: Yes

Reviewer #5: Yes

**********

3. Have the authors made all data underlying the findings in their manuscript fully available?

The PLOS Data policy

Reviewer #1: Yes

Reviewer #2: No

Reviewer #3: No

Reviewer #4: No

Reviewer #5: No

**********

4. Is the manuscript presented in an intelligible fashion and written in standard English?

Reviewer #1: Yes

Reviewer #2: Yes

Reviewer #3: Yes

Reviewer #4: Yes

Reviewer #5: Yes

**********

Reviewer #1: The manuscript is clearly written and thoughtfully organized, addressing a highly relevant and timely public health concern—the increasing rates of caesarean deliveries and their associated financial and quality-of-life implications. It provides meaningful insights into maternal health service utilization in India, with a particular focus on Tamil Nadu, where both institutional delivery and C-section rates are notably high. The study’s design, data collection procedures, and analytical approach are appropriate and well-articulated. Nonetheless, several revisions could further enhance the clarity, policy relevance, and overall scientific contribution of the paper.

• The manuscript is well written and addresses a relevant public health issue concerning the increasing prevalence of caesarean deliveries and their financial and quality-of-life implications.

• The study employs an appropriate cross-sectional design with well-defined objectives, contributing valuable evidence on maternal health outcomes in the Indian context.

• The sampling strategy is adequate; however, the rationale for selecting districts and the timeframe for data collection could be described more clearly.

• More information on participant recruitment and inclusion/exclusion criteria would enhance the transparency and reproducibility of the study.

• The authors should clarify how out-of-pocket expenditure (OOPE) was computed, specifying whether it included only direct medical costs or also indirect costs such as transport and income loss.

• The use of validated tools, such as the SF-12 for assessing health-related quality of life (HRQL), is appropriate, though providing reliability coefficients (e.g., Cronbach’s alpha) from the current dataset would strengthen the methodological rigor.

• The methods section could elaborate on how recall bias was minimized among postpartum women and whether any data verification procedures were applied uniformly across participants.

• The statistical approach, including logistic regression, is suitable for the study objectives; however, the inclusion criteria for variables in the multivariate model should be explained more explicitly.

• Confidence intervals and p-values should be reported consistently across all major findings to facilitate interpretation and comparison.

• The results are well presented and clearly highlight differences in OOPE and HRQL between caesarean and vaginal deliveries. A concise summary paragraph before the detailed tables could improve readability.

• Some tables, especially those presenting regression results, could be simplified by retaining only statistically significant variables or moved to supplementary material.

• The presentation of results could be enhanced by including a brief subgroup comparison (e.g., public vs. private hospitals) to illustrate variation in financial burden.

• The discussion should link the observed HRQL differences to potential underlying causes such as post-surgical pain, recovery duration, or socio-economic disparities.

• It would be useful to relate the findings to national benchmarks for catastrophic health expenditure to strengthen the policy implications.

• The data availability statement should be revised to comply fully with PLOS ONE’s open data policy, ensuring that replication of results is feasible.

• Minor language and formatting improvements (e.g., consistent decimal places, abbreviation use) would enhance the clarity and professional presentation of the manuscript.

Reviewer #2: The manuscript presents a well-conceived and policy-relevant study on the financial and quality-of-life impacts of Caesarean section (C-section) deliveries among postpartum women in Tamil Nadu, India. The topic is timely and significant from both public health and health economics perspectives. However, several methodological, analytical, and interpretive issues must be addressed before the paper can be considered for publication.

Major Issues

Novelty and Rationale: The introduction needs a clearer justification of the study’s unique contribution beyond existing work (e.g., Singh et al., 2023; Mishra & Mohanty, 2019). Authors should highlight what the primary data add compared with prior analyses of NFHS data.

Study Design: The rationale for selecting three districts and how they represent Tamil Nadu must be clarified. Provide details of the sampling approach, proportionality, and sample size calculation parameters.

Measurement: Discuss potential recall bias from self-reported data and describe SF-12 scoring, standardisation, and any validated Tamil version used.

Statistical Analysis: Provide justification for variable selection, address possible multicollinearity, and report model diagnostics (e.g., Hosmer–Lemeshow test). Consider sensitivity analyses for OOPE and ensure consistency in confidence interval presentation.

Results and Interpretation: Condense tables where possible, include mean OOPE differences with CIs, and interpret findings as associations rather than causal effects. Expand on policy implications, particularly regarding insurance schemes and maternal benefit programs.

Data Sharing: Comply with PLOS ONE’s open data requirements by depositing a de-identified dataset in a public repository.

Minor Issues

Correct typographical and grammatical errors (e.g., “finical” → “financial”) and ensure consistent terminology (“C-section” vs. “Caesarean section”).

Update older references and ensure all citations follow PLOS ONE formatting.

Expand the limitations section to discuss potential reporting bias, confounding, and geographic restriction.

Recommendation

Major Revision.

The study addresses an important research gap, but revisions are required to enhance methodological clarity, strengthen statistical rigor, and refine interpretation. Addressing these issues will considerably improve the manuscript’s quality and contribution to evidence on the economic and quality-of-life effects of C-section deliveries in India.

Reviewer #3: This manuscript addresses an important and policy relevant topic by examining the prevalence of caesarean section deliveries and their associations with out of pocket expenditure and health related quality of life among post partum mothers in Tamil Nadu, India. The study draws on primary data from multiple districts and applies appropriate descriptive and regression based analytical methods. The manuscript is generally well structured and clearly written. However, several methodological and interpretative issues, particularly related to the use of causal language, handling of the sampling design, and the strength of inferences drawn from cross sectional data, need to be addressed before the findings can be reliably interpreted and the manuscript considered for publication.

Abstract:

line 33: "indicators" instead of indications

line 43: Avoid causal language in a cross sectional study.

Example: High-risk pregnancies (AOR=15.90, 95% CI= 9.07-27.84, p<0.001), and private healthcare facilities (AOR= 3.03, 95% CI= 1.71-5.35, p<0.001) increased odds of C-sections.

Here the language is causal.

line 44-45: Please provide AOR with 95% CI for indebtedness and HRQL

Introduction:

line 67: add a coma after 2019-21.

Methods:

line 111-114: Sampling: Does the sample size calculation account for 20% non-response rate, or was it calculated manually? The sample size should be 480. I would also suggest to present the actual sample size first and then inflate with 20% non-response rate. Again, the authors report in introduction a 44.9% prevalence of C-section. Then why 50% estimate was used?

line 116: Given the use of a multistage cluster sampling design, the absence of a design effect in the sample size calculation is a major methodological omission. Ignoring clustering is likely to underestimate the required sample size and may compromise precision.

Line 118: Please explain MPI in-short here.

Line 127: If 20% non response rate was considered, why did the authors invite 510 mothers? It seems like the sample size was ~384. So 475 participants were not mandatory.

Was the reliability of the SF-12 questionnaire examined?

line 165: Please consider IBM SPSS (version 27).

Please explain, how variables for the adjusted model was selected? What were the variables adjusted for

Results:

Causal language should be avoided as this is a cross-sectional study. From this design, we can not claim any group membership had "higher" or "lower" odds of outcome. Rather the authors should frame their interpretation as "associated with higher/lower odds".

Table 4: Also report IQR

Table 6: Please recheck the title

Discussion:

Causal language should be avoided throughout the manuscript

Over interpretation of associations: Several explanations offered for the observed associations, for example profitability of private facilities, provider risk avoidance, fertility preferences, and medicolegal pressures, are speculative and not empirically examined in the study. While such interpretations may be plausible, they should be more clearly identified as hypotheses or contextual explanations rather than implied findings derived from the data.

Reviewer #4: 1. General Assessment & Relevance to PLOS ONE

Objectives

In this cross-sectional study, the project aims to assess the prevalence, indications, and effects of Caesarean section delivery on out-of-pocket expenditure and health-related quality of life of mothers in Tamil Nadu, a state in India. The problem described in this study is very important in a public health setting since the incidence of Caesarean delivery in developing countries—especially in Tamil Nadu—is alarmingly high.

The manuscript is largely well-structured, methodologically sound, and within the realm of PLOS ONE. The sampling methodology is robust, using valid tools (SF-12) appropriately, and statistics are properly conducted. Several very important methodological issues need to be clarified before a manuscript will be considered for publication.

2. Summary of Key Strengths

Timely and Important Topic: Theresearch records a proportionately very high Cesarean delivery rate of 52.6% and offers sound evidence of cost and medical repercussions in a state where such prevalence is common in India.

Strong Study Design: The application of multi-stage stratified cluster random sampling based on levels of MPI adds better representativeness and robustness to this study’s findings.

Outcome Measurement: A complete outcome measurement approach is achieved in this study, in which economic parameters (ooPE, distress financing) are appropriately linked with patient-reported outcomes (HRQL measured using SF-12).

Suitable Statistical Methodology: The application of Binary Logistic Regression analysis to calculate Adjusted Odds Ratios after controlling for variables in a suitable model.

Policy-Relevant Discussion: The discussion is effective in contextualizing research findings and developing a strategy with a multifaceted approach towards minimizing unnecessary Caesarean deliveries.

3. Major Concerns and Recommendations for Revision

A. Methodological Clarity and Limitations:

Study Design & Causality

The cross-sectional study design is appropriately cited as a shortcoming, but it ought to be further emphasized in this text that associations observed (e.g., from C-section to higher OOPE) are correlations and do not in any way prove causality. Thus, for example, HR pregnancy, a major precipitating factor for a C-section, can be a direct cause of increased expenditure and reduced HRQL.

Measurement and Classification:

High Risk Pregnancy & Complications: The important exposure variables are based solely on self-report by mothers and therefore are susceptible to recall and classification biases. Note will be given to this in future.

OOPE Categorization: Median split of OOPE with a cut-point at INR 10,000 is justified on grounds of skewness but leads to an overly simplistic treatment of a rather complex financial outcome. A rationale for this cut-point, perhaps in Methods, would be good.

SF-12 Scoring

The PCS and MCS scores were classified into "good" if they were 50 or higher and "poor" if they were less than 50 based on a cutoff of 50, which is based on the population mean. A brief mention of the validity of this cutoff in the post-partum population or an acknowledgment of this as a potential problem would improve this manuscript.

Potential Unmeasured Confounding: The regression models adjust for important socio-demographic and obstetric factors. Unmeasured factors such as preference for cesarean delivery by mother/physician, fear of litigation, or culture may affect both mode of delivery and outcomes. Such a situation will have to be noted in the limitations.

B. Presentation of Results:

Table 2 (Indications): The heading and format might be a bit confusing. Intra-partum complications are largely confined to C-sections in this table, with very minimal information on vaginal delivery. Perhaps a different heading for this table might clarify this impression, or an additional row indicating just how rare these complications are in vaginal delivery.

Clarity in Text: With regards to the results in the "Results" section, in stating the significance of the relationship between C-section and financial distress (p < 0.001), a clearer presentation would be to put it this way: "50.4% of mothers with C-section experienced distress financing, compared to 30.2% of mothers with vaginal delivery χ²=19.955, p<0.001."

Besides,

Table 5 Footnote:

This footnote mentions that "place of delivery was excluded from the model as it had strong multicollinearity with C-Section." This is a very important aspect which must also be highlighted in brief in "Methods" under "Analysis" in the main text.

C. Discussion and Interpretation:

**Contextualization of Findings:** The discourse can benefit from being linked to research literature in a more direct and specific manner, especially research literature referring to a similar Indian context. For instance, when referring to increased odds of C-section in a rural setting, research studies conducted in an Indian context identifying support or challenge to such a piece of information can be referred to.

Mechanisms Connecting C-section with HRQL: Elaborate on why C-section is linked to poor HRQL. Could it be attributed to a longer recovery time, pain associated with surgeries, psychosocial effects of such surgeries, or is it because of other variables?

Limitations Section: The existing limitations section is very short. It shall be enlarged to cover all issues in relation to:

The basic constraint of cross-sectional design for causal inference.

Recall vulnerability and self-reporting bias in important variables.

The possibility of residual confounding.

Generalizability: Although the sampling in three districts is quite strong, the very high rate of cesarean delivery might not be very representative of all the districts in Tamil Nadu.

4. Minor Corrections and Editorial Suggestions

Abstract: "finical impact" must be replaced with "financial impact".

Introduction: "C-Sections are rapidly raising" should be "rising".

Ways: Add the number of mothers who did not consent out of 510 approached to make it clearer.

Tables:

Table 1: Make sure the symbol for the conversion in US dollars (^#^) is placed in accordance with the header of the column. Table 1 & 4: Make sure all acronyms are properly explained in a table title or footnote, such as in Table 4 where PCS & MCS in the footnote, but inclusion of PCS & MCS in table 1/4 abbreviation list can help with clarity. References:

Please make sure all references follow PLOS ONE guidelines. Some references, such as Refs 3, 4, and 9, appear to be web citations or non-traditional sources, and their format can beverified. General Proofreading

A general proofreading of this work can help clarify typographical errors in some sentences. For instance, "We conducted this study i)" can be rewritten as "We conducted this study (i)".

5. Overall Recommendation Decision: Major Revision. The manuscript considers an important question in research and is well-designed, with important findings presented. However, in order for this manuscript to be publishable, it is important that the above-mentioned issues, especially concerning a proper explanation of methodology limitations, a better analytical presentation, and a better representation of research results, are given proper attention.

Reviewer #5: Caesarean Deliveries and their impact on financial burden and quality of life among

post-partum mothers in Tamil Nadu, India

The study examines a significant public health concern—the rise in Caesarean sections (C-sections) in India—and offers significant empirical evidence on their economic implications and effects on quality of life. Nevertheless, substantial methodological and data-sharing issues need to be resolved prior to its publication.

The C-section rate of 52.6% reported in this study substantially exceeds the WHO recommendation of 10-15%. Examining the factors contributing to and the implications of this trend in Tamil Nadu is significant. Employing the SF-12 survey for quality-of-life evaluation and binary logistic regression for analysis offers a robust quantitative basis.

I would suggest a substantial revision. The comments are presented as follows:

1.Since 60% of mothers delivered within six months prior to the survey and others within up to a year, there is potential for recall bias regarding specific health complications and expenditures. The authors should explicitly address how they mitigated this issue beyond merely "verification with bills," especially concerning the qualitative components of the SF-12 survey.

2.The study indicates that mothers residing in rural areas are more likely to have cesarean sections. This contradicts traditional patterns and warrants a more thorough examination of whether it is attributable to insufficient emergency obstetric services or to the practice of "defensive medicine" in rural healthcare facilities.

3.The authors contacted 510 mothers to obtain a final sample of 475, with many exclusions resulting from refusals to provide consent. Including a concise non-response analysis (e.g., comparing the fundamental demographics of individuals who declined participation with those who participated) would assist in demonstrating that your final sample is free from bias.

4.Over sixty percent of mothers delivered within six months of the survey, although some were up to a year postpartum. The recall of precise OOPE may diminish over time. The authors confirmed details by cross-referencing hospital bills and discharged summaries in some instances; however, they should explicitly specify the proportion of the sample for which tangible documents were available for verification, compared with those who relied exclusively on recollection.

5.In the logistic regression models, the authors incorporated several independent variables, including district, affluence index, education level, and site of delivery. It is advisable to report Variance Inflation Factors (VIF) or other diagnostic assessments to verify that these variables, particularly wealth and education, are not highly correlated, as such correlation could distort your Adjusted Odds Ratios (AOR).

6.A mother was classified as "high-risk" if she reported at least one indication. However, "prior cesarean section" was a notable indication, accounting for 41.2%. Since a previous C-section almost always leads to a repeat C-section in many clinical settings, it may be beneficial to run a sensitivity analysis excluding these cases to see if the other risk factors (like gestational diabetes or pre-eclampsia) still hold the same weight.

7.Quality-of-life scores (SF-12) may vary considerably between one month and twelve months postpartum. It is advisable to incorporate "time since delivery" as a covariate in your regression models for PCS and MCS to ensure that the observed "poor" quality of life is genuinely linked to the mode of delivery rather than merely the recency of childbirth.

**********

what does this mean?). If published, this will include your full peer review and any attached files.

If you choose “no”, your identity will remain anonymous but your review may still be made public.

**Do you want your identity to be public for this peer review?**  For information about this choice, including consent withdrawal, please see our Privacy Policy

Reviewer #1: **Yes:** Mohsin Hassan Alvi

Reviewer #2: **Yes:** Montasir Ahmed

Reviewer #3: No

Reviewer #4: No

Reviewer #5: No

**********

[NOTE: If reviewer comments were submitted as an attachment file, they will be attached to this email and accessible via the submission site. Please log into your account, locate the manuscript record, and check for the action link "View Attachments". If this link does not appear, there are no attachment files.]

To ensure your figures meet our technical requirements, please review our figure guidelines: https://journals.plos.org/plosone/s/figures

You may also use PLOS’s free figure tool, NAAS, to help you prepare publication quality figures: https://journals.plos.org/plosone/s/figures#loc-tools-for-figure-preparation.

NAAS will assess whether your figures meet our technical requirements by comparing each figure against our figure specifications.

Attachment Submitted filename: C-section and HRQL in India.pdf

# Author comment: Author response to Decision Letter 1

**DOI:** 10.1371/journal.pone.0358778.r002

19 Mar 2026

Point by point response to review comments

Respected Editor and Reviewers,

Thank you for the careful and thorough review of our manuscript. We sincerely appreciate the insightful comments and suggestions provided. We have carefully considered each comment and have revised the manuscript accordingly.

We hereby submit a detailed point-by-point response to the reviewers’ comments for your kind consideration. The reviewer comments have been highlighted, and our responses are provided alongside them, with corresponding line numbers indicating where revisions have been made in the manuscript.

In compliance with the data-sharing requirements of PLOS ONE, we are also submitting the de-identified microdata as a supplementary file. Additionally, we have included both a revised manuscript with tracked changes and a clean version of the manuscript, along with the required supporting documents, for your review and consideration.

Sincerely

Corresponding Author

Editor Comments

1.Please ensure that your manuscript meets PLOS ONE's style requirements, including those for file naming. The PLOS ONE style templates can be found at

https://journals.plos.org/plosone/s/file?id=wjVg/PLOSOne_formatting_sample_main_body.pdf and

https://journals.plos.org/plosone/s/file?id=ba62/PLOSOne_formatting_sample_title_authors_affiliations.pdf

Author response: Thank you we have ensured to submit the manuscript inline with the PLOS ONE Style requirements.

2. PLOS requires an ORCID iD for the corresponding author in Editorial Manager on papers submitted after December 6th, 2016. Please ensure that you have an ORCID iD and that it is validated in Editorial Manager. To do this, go to ‘Update my Information’ (in the upper left-hand corner of the main menu), and click on the Fetch/Validate link next to the ORCID field. This will take you to the ORCID site and allow you to create a new iD or authenticate a pre-existing iD in Editorial Manager.

Author response: Thank you the ORCID iD of the corresponding author was linked to the editorial manager.

3. In the online submission form, you indicated that “The study data will be made available to individual researchers/institutions upon a reasonable request to the corresponding author.”

All PLOS journals now require all data underlying the findings described in their manuscript to be freely available to other researchers, either 1. In a public repository, 2. Within the manuscript itself, or 3. Uploaded as supplementary information.

This policy applies to all data except where public deposition would breach compliance with the protocol approved by your research ethics board. If your data cannot be made publicly available for ethical or legal reasons (e.g., public availability would compromise patient privacy), please explain your reasons on resubmission and your exemption request will be escalated for approval.

Author response: Respected editor, in compliance with the PLOS ONE data sharing requirement, we have uploaded the data as supplementary information. The data is in .xls format accessible using Microsoft Excel.

4. If the reviewer comments include a recommendation to cite specific previously published works, please review and evaluate these publications to determine whether they are relevant and should be cited. There is no requirement to cite these works unless the editor has indicated otherwise.

Author response: Respected editor, thank you for the suggestion. We have reviewed the previously published works quoted by the reviewers and cited the ones which were appropriate for our work.

Reviewer Comments

Comments to the Author

1. Is the manuscript technically sound, and do the data support the conclusions?

The manuscript must describe a technically sound piece of scientific research with data that supports the conclusions. Experiments must have been conducted rigorously, with appropriate controls, replication, and sample sizes. The conclusions must be drawn appropriately based on the data presented.

Reviewer #1: Yes

Reviewer #2: Yes

Reviewer #3: Partly

Reviewer #4: Partly

Reviewer #5: Yes

________________________________________

2. Has the statistical analysis been performed appropriately and rigorously?

Reviewer #1: Yes

Reviewer #2: Yes

Reviewer #3: Yes

Reviewer #4: Yes

Reviewer #5: Yes

________________________________________

3. Have the authors made all data underlying the findings in their manuscript fully available?

The PLOS Data policy requires authors to make all data underlying the findings described in their manuscript fully available without restriction, with rare exception (please refer to the Data Availability Statement in the manuscript PDF file). The data should be provided as part of the manuscript or its supporting information, or deposited to a public repository. For example, in addition to summary statistics, the data points behind means, medians and variance measures should be available. If there are restrictions on publicly sharing data—e.g. participant privacy or use of data from a third party—those must be specified.

Reviewer #1: Yes

Reviewer #2: No

Reviewer #3: No

Reviewer #4: No

Reviewer #5: No

________________________________________

4. Is the manuscript presented in an intelligible fashion and written in standard English?

PLOS ONE does not copyedit accepted manuscripts, so the language in submitted articles must be clear, correct, and unambiguous. Any typographical or grammatical errors should be corrected at revision, so please note any specific errors here.

Reviewer #1: Yes

Reviewer #2: Yes

Reviewer #3: Yes

Reviewer #4: Yes

Reviewer #5: Yes

________________________________________

5. Review Comments to the Author

Please use the space provided to explain your answers to the questions above. You may also include additional comments for the author, including concerns about dual publication, research ethics, or publication ethics. (Please upload your review as an attachment if it exceeds 20,000 characters)

Reviewer #1: The manuscript is clearly written and thoughtfully organized, addressing a highly relevant and timely public health concern—the increasing rates of caesarean deliveries and their associated financial and quality-of-life implications. It provides meaningful insights into maternal health service utilization in India, with a particular focus on Tamil Nadu, where both institutional delivery and C-section rates are notably high. The study’s design, data collection procedures, and analytical approach are appropriate and well-articulated. Nonetheless, several revisions could further enhance the clarity, policy relevance, and overall scientific contribution of the paper.

• The manuscript is well written and addresses a relevant public health issue concerning the increasing prevalence of caesarean deliveries and their financial and quality-of-life implications.

• The study employs an appropriate cross-sectional design with well-defined objectives, contributing valuable evidence on maternal health outcomes in the Indian context.

Author response: Respected reviewer, thank you for the meticulous review of our manuscript. We have revised our manuscript in accordance with the suggestions made. We believe that we have satisfactorily addressed the comments. We look forward to your positive consideration of our work.

• The sampling strategy is adequate; however, the rationale for selecting districts and the timeframe for data collection could be described more clearly.

Author response: Respected reviewer, thank you for the comment. We have revised the methods section of our manuscript to provide a clearer detail on timeframe for data collection and rationale for selecting districts. The corresponding changes were made in line 102-103 and line 119-124 respectively.

• More information on participant recruitment and inclusion/exclusion criteria would enhance the transparency and reproducibility of the study.

Author response: Respected reviewer, we have provided a detailed account of participant recruitment reporting the inclusion and exclusion criteria. The corresponding revisions were made in Line 134-138

• The authors should clarify how out-of-pocket expenditure (OOPE) was computed, specifying whether it included only direct medical costs or also indirect costs such as transport and income loss.

Author response: Respected reviewer, thank you for the comment. We have captured OOPE as a combination of direct and in-direct costs. The direct costs included OOPE paid for hospital stay, tests, medicines and other expenses; while indirect costs included transportation, paid-caregivers, food & accommodation. The details of the same were provided in Line 153-158 of the revised manuscript.

• The use of validated tools, such as the SF-12 for assessing health-related quality of life (HRQL), is appropriate, though providing reliability coefficients (e.g., Cronbach’s alpha) from the current dataset would strengthen the methodological rigor.

Author response: respected reviewer thank you for the suggestion. We have provided the details of Cronbach’s alpha for SF-12 based on current dataset. The details are provided in Line 166-167.

• The methods section could elaborate on how recall bias was minimized among postpartum women and whether any data verification procedures were applied uniformly across participants.

Author response: Respected reviewer, thank you for the comment. We recognize the potential for recall bias in the study variables like out-of-pocket expenses and high-risk pregnancies. We verified the high-risk pregnancies based on the MCP card and hospital discharge summaries that all mothers with institutional deliveries report. Further, the OOPE while self-reported was captured as a composite variable of direct and indirect costs. We have reported the data verification procedures in the methods section (Line 156-158) and the potential for recall bias as the study limitation (Line 389-391)

• The statistical approach, including logistic regression, is suitable for the study objectives; however, the inclusion criteria for variables in the multivariate model should be explained more explicitly.

Author response: Respected reviewer, thank you for the suggestion. The independent variables in the multi-variate analysis were included based on prior published literature and the bivariate results observed in this study. We have clarified the same in the revised manuscript in lines 208-212

• Confidence intervals and p-values should be reported consistently across all major findings to facilitate interpretation and comparison.

Author response: Respected reviewer, thank you. We have consistently reported the CI and p-values across all major findings.

• The results are well presented and clearly highlight differences in OOPE and HRQL between caesarean and vaginal deliveries. A concise summary paragraph before the detailed tables could improve readability.

Author response: Respected reviewer, thank you for the suggestion. We have provided a concise summary of the findings before the detailed tables. The edits were made to the following sections of the revised manuscript in lines 285-295, and lines 300-305.

• Some tables, especially those presenting regression results, could be simplified by retaining only statistically significant variables or moved to supplementary material.

Author response: Respected reviewer, thank you. We have reported the significant results in our regression tables. The full tables were moved to supplementary files.

• The presentation of results could be enhanced by including a brief subgroup comparison (e.g., public vs. private hospitals) to illustrate variation in financial burden.

Author response: Respected reviewer, thank you for the suggestion. We have included a brief sub-group comparison of public vs. private hospitals to illustrate variation in financial burden. Since this was not our primary objective these results were provided as Supplementary file 1 table 8.

• The discussion should link the observed HRQL differences to potential underlying causes such as post-surgical pain, recovery duration, or socio-economic disparities.

Author response: Respected reviewer, thank you for the suggestion. We have revised our introduction section to link the observed HRQL differences to underlying causes like post-surgical pain, recovery duration, limited functionality etc. The revisions were made in lines 356-358.

• It would be useful to relate the findings to national benchmarks for catastrophic health expenditure to strengthen the policy implications.

Author response: Respected reviewer, thank you for the we have related our estimates to recent National Family Health Survey estimates of India. The revision was made in line 353-355.

• The data availability statement should be revised to comply fully with PLOS ONE’s open data policy, ensuring that replication of results is feasible.

Author response: Respected reviewer, thank you. We have revised our data availability statement to comply with the PLOS ONE’s open data policy. The data was provided as a supplementary file.

• Minor language and formatting improvements (e.g., consistent decimal places, abbreviation use) would enhance the clarity and professional presentation of the manuscript.

Author response: Respected reviewer, thank you for the comment. We have made the revisions to improve language and formatting of our manuscript.

Reviewer 2

Reviewer #2: The manuscript presents a well-conceived and policy-relevant study on the financial and quality-of-life impacts of Caesarean section (C-section) deliveries among postpartum women in Tamil Nadu, India. The topic is timely and significant from both public health and health economics perspectives. However, several methodological, analytical, and interpretive issues must be addressed before the paper can be considered for publication.

Major Issues

Novelty and Rationale: The introduction needs a clearer justification of the study’s unique contribution beyond existing work (e.g., Singh et al., 2023; Mishra & Mohanty, 2019). Authors should highlight what the primary data add compared with prior analyses of NFHS data.

Author response: Respected reviewer thank you for the suggestion, we have clarified the justification for our study citing what previous studies like Singh et al., 2023 and Mishra & Mohanty, 2019 reported; and what more our study adds. The revisions were made in the lines 83-88 and 91-94.

Study Design: The rationale for selecting three districts and how they represent Tamil Nadu must be clarified. Provide details of the sampling approach, proportionality, and sample size calculation parameters.

Author response: Respected reviewer, thank you for the comment. We have provided the details of sample size parameters [lines 112-116], Sampling approach and rationale for selecting the three districts [lines 120-124] in the methods section.

Measurement: Discuss potential recall bias from self-reported data and describe SF-12 scoring, standardisation, and any validated Tamil version used.

Author response: Respected reviewer, thank you for the suggestion we have discussed the potential for recall bias from self-reported data. We have also described SF-12 scoring standardization. We could not find a separate validated Tamil version. These were duly recognized as limitations of the study in line 389-393

Statistical Analysis: Provide justification for variable selection, address possible multicollinearity, and report model diagnostics (e.g., Hosmer–Lemeshow test). Consider sensitivity analyses for OOPE and ensure consistency in confidence interval presentation.

Author response: We thank the reviewer for this valuable comment. Independent variables included in the multivariable models were selected based on prior literature and statistically significant associations observed in bivariate analyses (Lines 208-212).

We assessed multicollinearity using the Variance Inflation Factor (VIF), and no evidence of problematic multicollinearity was observed (Lines 214-215).

Model diagnostics were performed using the Hosmer–Lemeshow goodness-of-fit test, and the results have been reported with the corresponding regression tables.

To ensure robustness of findings, we conducted sensitivity analyses for the outcomes by excluding previous history of C-section from the indications of high-risk pregnancy. The results of sensitivity analysis were provided in the supplementary files. Additionally, standard errors were adjusted using a clustered robust approach to account for potential intra-cluster correlation.

We have also ensured consistency in the reporting of confidence intervals throughout the manuscript.

Results and Interpretation: Condense tables where possible, include mean OOPE differences with CIs, and interpret findings as associations rather than causal effects. Expand on policy implications, particularly regarding insurance schemes and maternal benefit programs.

Author response: Respected reviewer, thank you for the comments. We have condensed our tables by reporting only significant observations [Table 3, 5, 6]. We have provided the mean OOPE differences with 95% CI estimates [Table 4]. We have interpreted our findings as associations and strengthened our discussion section to expand on policy implications [lines 376-387].

Data Sharing: Comply with PLOS ONE’s open data requirements by depositing a de-identified dataset in a public repository.

Author response: Respected reviewer, thank you. We have revised our data availability statement to comply with the PLOS ONE’s open data policy. We have provided the dataset as supplementary file w.

Minor Issues

Correct typographical and grammatical errors (e.g., “finical” → “financial”) and ensure consistent terminology (“C-section” vs. “Caesarean section”).

Author response: Thank you we have corrected all the typographical and grammatical errors.

Update older references and ensure all citations follow PLOS ONE formatting.

Author response: Thank you for the suggestion we have ensured to update the references and all citations follow PLOS ONE formatting.

Expand the limitations section to discuss potential reporting bias, confounding, and geographic restriction.

Author response: We thank the reviewer for the suggestions. We have expanded the limitations section to report the non-causal nature of study design, self-reported responses, social desirability bias, unmeasured confounding and limited geographical coverage. The revisions were made in lines 388-401 of the revised manuscript.

Recommendation

Major Revision.

The study addresses an important research gap, but revisions are required to enhance methodological clarity, strengthen statistical rigor, and refine interpretation. Addressing these issues will considerably improve the manuscript’s quality and contribution to evidence on the economic and quality-of-life effects of C-section deliveries in India.

Author response: Respected reviewer, thank you for the meticulous review of our manuscript. We have revised our manuscript in accordance with the suggestions made. We believe that we have satisfactorily addressed the comments. We look forward to your positive consideration of our work.

Reviewer 2- Attached file

This is a well-conceived and relevant study that addresses a timely and important issue in maternal health in India—the growing prevalence of Caesarean section (C-section) deliveries and their financial and quality-of-life implications. The topic is significant from both public health and health economics perspectives, and the paper provides useful evidence from a state known for its high institutional delivery rates. However, there are several methodological, analytical, and interpretive concerns that need to be addressed before the paper can be considered for publication.

Major Comments

Study Rationale and Novelty

• The introduction establishes context but needs a stronger justification of the study’s novel contribution. Similar studies have been conducted in Indian states using NFHS data. The authors should clarify how their primary data collection adds value (e.g., inclusion of HRQL outcomes, district-level insights, or cost breakdowns).

• Please clarify how this study adds to prior work by Singh et al. (2023, BMC Health Services Research) and Mishra & Mohanty (2019, Int J Equity Health), both of which examined OOPE for C-sections in India.

Author response: Respected reviewer thank you for the suggestion, we have clarified the justification for our study citing what previous studies like Singh et al., 2023 and Mishra & Mohanty, 2019 reported; and what more our study adds. The revisions were made in the lines 83-88 and 91-94.

Study Design and Sampling

• The sampling strategy needs clearer explanation. Although a multistage stratified cluster random sampling is described, the rationale for selecting only three districts may introduce selection bias. Please justify how these districts represent Tamil Nadu overall.

• Clarify whether the sampling was proportional to population size.

• The sample size calculation should explicitly mention the formula used and parameters (confidence interval, expected prevalence, precision, non-response rate).

Author response: Respected reviewer, thank you for the comment. We have provided the details of sample size parameters [lines 112-116], Sampling approach and rationale for selecting the three districts [lines 119-126] in the methods section.

We have provided the formula used in line 112

Measurement and Data Collection

• The use of self-reported data for C-section and OOPE introduces potential recall bias, particularly among mothers who delivered up to a year prior. Discuss this limitation.

• The SF-12 tool is appropriate but please specify scoring and cut-off criteria (e.g., how PCS and MCS were standardized to a mean of 50). Reference to the validated Tamil version, if available, would strengthen credibility.

Author response: Respected reviewer, thank you for the suggestion we have discussed the potential for recall bias from self-reported data. We have also described SF-12 scoring standardization. We could not find a separate validated Tamil version. These were duly recognized as limitations of the study in line 391-393

Statistical Analysis

• The choice of logistic regression is sound; however, the inclusion/exclusion of variables needs clearer justification. Were collinearity diagnostics performed?

Author response: Respected reviewer, thank you for the query. We have included the variables based on published literature and bivariate associations. Multicollinearity was testing using variance inflation factor. We have provided the details of VIF for each logistic regression model in the corresponding regression tables’ foot notes.

• OOPE was dichotomized using the median, but a sensitivity analysis (e.g., log-transformed continuous outcome) could provide additional robustness.

Author response: Respected reviewer, thank you for the suggestion. We have conducted the sensitivity analysis by log transforming the OOPE. We obtained similar associations in our sensitivity analysis. The corresponding results were provided in supplementary file 1, table 6.

• In Table 5, the inclusion of district, education, and wealth index together with mode of delivery may cause multicollinearity; explain variance inflation factors or other checks.

Author response: Respected reviewer, thank you for the suggestion. We have provided the details of the VIF checks in line 218-220.

• In table 2, it requires consistency for presenting CI, not like 0.4 (0.00-1.9)

Author response: Respected reviewer, thank you for the suggestion. We have made the suggested revision.

• The authors should report model goodness-of-fit statistics (e.g., Hosmer-Lemeshow test) in the results narrative.

Author response: Respected reviewer, thank you for the suggestion, we have reported the goodness of fit statistics in the results narrative.

Results Presentation

• Tables are comprehensive but could be condensed. Consider moving descriptive details (e.g., high-risk pregnancy indicators) to supplementary material.

• It would strengthen the paper to present mean OOPE by delivery mode and effect sizes (mean differences with confidence intervals), not only odds ratios.

Author response: Respected reviewer, thank you for the suggestion. We have condensed our logistic regression tables (tables 3, 5 and 6) to report only significant findings. The complete tables were reported in supplementary file 1. We could not move Table 2 to supplementary file since moving it would require restricting the results and discussion sections.

We have improved the presentation of table 5 by reporting mean and 95% CI of OOPE across delivery mode and other independent variables.

Interpretation and Discussion

• The discussion appropriately references prior literature, but the policy implications section could be expanded. For example, discuss how insurance schemes (like PM-JAY) or maternal benefit programs might mitigate financial distress.

• Some statements imply causality (“C-section reduces HRQL”) despite cross-sectional data — please rephrase these as associations, not effects.

• Include potential confounders such as type of facility (public/private), maternal complications, or socioeconomic status in the causal discussion.

Author response: Respected reviewer, thank you for the suggestions on interpretation and discussion section. We have strengthened our discussion to include policy implications of the study in lines 376-387.

We have discussed in detail the potential confounders including socio-economic status, maternal complications, and place of delivery in lines 318-320; lines 327-333; and lines 336-349.

We have ensured to refrain from reporting our findings as causal association.

Data Availability and Ethical Statement

• The current data availability statement (“available upon reasonable request”) does not fully comply with PLOS ONE’s open data policy. The dataset (de-identified) should be deposited in a public repository if possible.

Author response: Respected reviewer, thank you. We have provided deidentified data as a supporting information.

Minor Comments

Abstract:

• Please report the study period (April–June 2023) in the Methods section of the abstract.

• The abstract is concise but contains typographical errors (“finical” → “financial”).

Author response: Respected reviewer, thank you for the suggestions. We have made the suggested revisions in our Abstract.

Writing and Formatting

• Some typographical and grammatical inconsistencies (e.g., spacing after periods, capitalization of “C-section”) need minor proofreading.

• Ensure consistent use of “C-section” versus “Caesarean section”.

Author response: Thank you we have corrected all the typographical and grammatical errors.

References:

• Ensure all references are formatted according to PLOS ONE guidelines.

• Some references (e.g., 4, 5, 6) appear to cite websites; ensure all are correctly formatted according to PLOS ONE style.

• References are comprehensive, though some older citations could be replaced with more recent evidence (2023–2024) where available.

Author response: Thank you for the suggestion we have ensured to update the references and all citations follow PLOS ONE formatting.

Limitations

• The limitations section should be expanded to discuss: (i) potential reporting bias, (ii) residual confounding, and (iii) the restriction to only three districts.

Author response: We thank the reviewer for the suggestions. We have expanded the limitations section to report the non-causal nature of study design, self-reported responses, social desirability bias, unmeasured confounding and limited geographical coverage. The revisions were made in lines 388-401 of the revised manuscript.

Recommendation

Major Revision

The manuscript presents valuable findings with strong public health relevance but requires improvements in methodological clarity, statistical robustness, and cautious interpretation. Addressing the above concerns will substantially strengthen the paper’s contribution to the evidence base on the economic and quality-of-life implications of C-section deliveries in India.

Author response: Respected reviewer, thank you for the meticulous review of our manuscript. We have revised our manuscript in accordance with the suggestions made. We believe that we have satisfactorily addressed the comments. We look forward to your positive consideration of our work.

Reviewer 3

Reviewer #3: This manuscript addresses an important and policy relevant topic by examining the prevalence of caesarean section deliveries and their associations with out of pocket expenditure and health related quality of life among post partum mothers in Tamil Nadu, India. The study draws on primary data from multiple districts and applies appropriate descriptive and regression based analytical methods. The manuscript is generally well structured and clearly written. However, several methodological and interpretative issues, particularly related to the use of causal language, handling of the sampling design, and the strength of inferences drawn from cross sectional data, need to be addressed before the findings can be reliably interpreted and the manuscript considered for publication.

Abstract:

line 33: "indicators" instead of indications

line 43: Avoid causal language in a cross sectional study.

Example: High-risk pregnancies (AOR=15.90, 95% CI= 9.07-27.84, p<0.001), and private healthcare facilities (AOR= 3.03, 95% CI= 1.71-5.35, p<0.001) increased odds of C-sections.

Here the language is causal.

line 44-45: Please provide AOR with 95% CI for indebtedness and HRQL

Author response: Respected reviewer, thank you for the comments. We had revised our abstract to reflect on the suggested changes.

Introduction:

line 67: add a coma after 2019-21.

Author response: Thank you we me made the suggested edit.

Methods:

line 111-114: Sampling: Does the sample size calculation account for 20% non-response rate, or was it calculated manually? The sample size should be 480. I would also suggest to present the actual sample size first and then inflate with 20% non-response rate. Again, the authors report in introduction a 44.9% prevalence of C-section. Then why 50% estimate was used?

Author response: Respected reviewer, thank you for the suggestion we have used an estimate of 50% for C-section deliveries given that

Respected reviewer thank you for the comment. While the overall prevalence of C-section in Tamil Nadu was 44.9% (according to NFHS-5), the C-section prevalence varied among the three districts from 43.1% to 67.6%. Hence we preferred to take an estimate of 50%. Additionally, we would like to submit that the difference in the sample estimate with p=44.9% and p=50% was not substantial, and was covered in the sample that we obtained extra. We have provided a detailed account of our sample size estimation in lines 112-116.

With respect to sample size estimation, our original sample size was 384, which was inflated the sample to 460 (accounting for an non-response rate of 20%) and rounded off the sample to a target sample of 475.

We did not account for design effect in our sample size. This is a major limitation which we have recognized in our limitations section.

line 116: Given the use of a multistage cluster sampling design, the absence of a design effect in the sample size calculation is a major methodological omission. Ignoring clustering is likely to underestimate the required sample size and may compromise precision.

Author response: We thank the reviewer for highlighting this important point. We acknowledge that a design effect was not incorporated in the initial sample size calculation despite the use of a multistage cluster sampling design, which may have implications for the precision of the estimates. This has now been acknowledged as a limitation in the revised manuscript (line 399-401).

To account for the clustering in the data during the analytical stage, we adjusted the standard errors using a clustered robust variance estimator in Stata, which helps address potential intra-cluster correlation and provides more reliable inference.

Line 118: Please explain MPI in-short here.

Author response: Respected reviewer, thank you for the suggestion we have provided the details about MPI. The revisions were made in lines 119-124 of the revised manuscript.

Line 127: If 20% non response rate was considered, why did the authors invite 510 mothers? It seems like the sample size was ~384. So 475 participants were not mandatory.

Author response: Respected reviewer, thank you for raising the point. Yes, 475 were not mandatory we have inflated the sample size from 384 to 460 by applying a 20% non-response rate and further rounded the sample size to a target sample of 475. We have provided the clarification of the same in our revised version in the lines 112-116.

Was the reliability of the SF-12 questionnaire examined?

Author response: Respected reviewer, thank you for pointing at the reliability estimate. We computed Cronbach’s Alpha for SF-12 and observed it to be 0.85. We have reported the same in our revised manuscript in line 167.

line 165: Please consider IBM SPSS (version 27).

Please explain, how variables for the adjusted model was selected? What were the variables adjusted for

Author response: Thank you for the suggestion. We have provided the details of what independent variables were adjusted for in the multivariate model, how they have been identified. The revisions were made to line 180-182

Results:

Causal language should be avoided as this is a cross-sectional study. From this design, we can not claim any group membership had "higher" or "lower" odds of outcome. Rather the authors should frame their interpretation as "associated with higher/lower odds".

Author response: Respected reviewer, thank you for the comment, we have revised our results and discussion section interpreting the findings as association.

Table 4: Also report IQR

Author response: Thank you we have provided the IQR in table 4

Table 6: Please recheck the title

Author response: Thank you we have revised the title of Table 6. The revised title is “Factors associated with Health-Related Quality of Life: the role of mode of delivery (n=475)”

Discussion:

Causal language should be avoided throughout the manuscript

Over interpretation of associations: Several explanations offered for the observed associations, for example profitability of private facilities, provider risk avoidance, fertility preferences, and medicolegal pressures, are speculative and not empirically examined in the study. While such interpretations may be plausible, they should be more clearly identified as hypotheses or contextual explanations rather than implied findings derived from the data.

Author response: We thank the reviewer for this important suggestion. We have carefully revised the manuscript to avoid causal language and ensure that the findings are presented as associations rather than causal relationships. In addition, statements that could be interpreted as over-interpretation have been modified. The discussion section has been revised accordingly to better reflect the observational nature of the study.

Reviewer 4

Reviewer #4: 1. General Assessment & Relevance to PLOS ONE

Objectives

In this cross-sectional study, the project aims to assess the prevalence, indications, and effects of Caesarean section delivery on out-of-pocket expenditure and health-related quality of life of mothers in Tamil Nadu, a state in India. The problem described in this study is very important in a public health setting since the incidence of Caesarean delivery in developing countries—especially in Tamil Nadu—is alarmingly high.

The manuscript is largely well-structured, methodologically sound, and within the realm of PLOS ONE. The sampling methodology is robust, using valid tools (SF-12) appropriately, and statistics are properly conducted. Several very important methodological issues need to be clarified before a manuscript will be considered for publication.

2. Summary of Key Strengths

Timely and Important Topic: Theresearch records a proportionately very high Cesarean delivery rate of 52.6% and offers sound evidence of cost and medical repercussions in a state where such prevalence is common in India.

Strong Study Design: The application of multi-stage stratified cluster random sampling based on levels of MPI adds better representativeness and robustness to this study’s findings.

Outcome Measurement: A complete outcome measurement approach is achieved in this study, in which economic parameters (ooPE, distress financing) are appropriately linked with patient-reported outcomes (HRQL measured using SF-12).

Suitable Statistical Methodology: The application of Binary Logistic Regression analysis to calculate Adjusted Odds Ratios after controlling for variables in a suitable model.

Policy-Relevant Discussion: The discussion is effective in contextualizing research findings and developing a strategy with a multifaceted approach towards minimizing unnecessary Caesarean deliveries.

Author response: We sincerely thank the reviewer for the positive and encouraging comments on our manuscript. We appreciate the recognition of the study’s relevance in examining the high prevalence of caesarean section deliveries and their associated economic and health implications in Tamil Nadu. We are grateful for the reviewer’s thoughtful observations and have provided a detailed point-by-point response to the comments below. We hope that the revisions and clarifications adequately address the reviewer’s suggestions and look forward to the favorable consideration of our revised manuscript.

3. Major Concerns and Recommendations for Revision

A. Methodological Clarity and Limitations:

Study Design & Causality

The cross-sectional study design is appropriately cited as a shortcoming, but it ought to be further emphasized in this text that associations observed (e.g., from C-section to higher OOPE) are correlations and do not in any way prove causality. Thus, for example, HR pregnancy, a major precipitating factor for a C-section, can be a direct cause of increased expenditure and reduced HRQL.

Author response: Respected reviewer, thank you for the suggestion. We have revised our limitations section, to indicate that the observed associations are correlational and do not infer causality.

Measurement and Classification:

High Risk Pregnancy & Complications: The important exposure variables are based solely on self-report by mothers and therefore are susceptible to recall and classification biases. Note will be given to this in future.

Author response: Respected reviewer, thank you for the comment. We have reported the potential for recall bias as a limitation of our study. Additionally, we have clarified in our methods section how recall bias for variables like high-risk pregnancy indications and intra partum complications were minimized.

OOPE Categorization: Median split of OOPE with a cut-point at INR 10,000 is justified on grounds of skewness but leads to an overly simplistic treatment of a rather complex financial outcome. A rationale for this cut-point, perhaps in Methods, would be good.

Author response: Respected reviewer, thank you for the suggestion. Previous studies on OOPE for Institutional deliveries in Indian settings and in Tamil Nadu has reported average OOPE to extent of INR.10000/-, thus we used INR.10000/- as a cutoff in our study. We have provided a clarification of this in our revised manuscript, providing a detailed description and citing relevant work [line 182-186].

SF-12 Scoring

The PCS and MCS scores were classified into "good" if they were 50 or higher and "poor" if they were less than 50 based on a cutoff of 50, which is based on the population mean. A brief mention of the validity of this cutoff in the post-partum population or an acknowledgment of this as a potential problem would improve this manuscript.

Author response: Respected reviewer, thank you for the comment. We agree with your suggestion and has reported the validity of the cutoff as a limitation. The revisions were made to lines 391-393

Potential Unmeasured Confounding: The regression models adjust for important socio-demographic and obstetric factors. Unmeasured factors such as preference for cesarean delivery by mother/physician, fear of litigation, or culture may affect both mode of delivery and outcomes. Such a situation will have to be noted in the limitations.

Author response: Respected reviewer, thank you for the suggestion. We have strengthened our limitations sections by recognizing the said limitations of our study. The revisions were made to the following sections of the revised manuscript- lines 393-396.

B. Presentation of Results:

Table 2 (Indications): The heading and format might be a bit confusing. Intra-partum complications are largely confined to C-sections in this table, with very minimal information on vaginal delivery. Perhaps a different heading for this table might clarify this impression, or an additional row indicating just how rare these complications are in vaginal delivery.

Author response: Respected reviewer, thank you for the comment. We have revised the title of Table 2 to providing clarification. The revised title for table 2 is “Distribution of High-Risk Pregnancy Indications and Intrapartum Complications by Mode of Delivery”

Clarity in Text: With regards to the results in the "Results" section, in stating the significance of the relationship between C-section and financial distress (p < 0.001), a clearer presentation would be to put it this way: "50.4% of mothers with C-section experienced distress financing, compared to 30.2% of mothers with vaginal delivery χ²=19.955, p<0.001."

Besides,

Author response: Respected reviewer, thank you. We have made the suggested change to improve clarity in text.

Table 5 Footnote:

This footnote mentions that "place of delivery was excluded from the model as it had strong multicollinearity with C-Section." This is a very important aspect which must also be highlighted in brief in "Methods" under "Analysis" in the main text.

Author response: Respected reviewer, thank you for the comment. We have made the suggested changes to the methods section.

C. Discussion and Interpretation:

**Contextualization of Findings:** The discourse can benefit from being linked to research literature in a more direct and specific manner, especially research literature referring to a similar Indian context. For instance, when referring to increased odds of C-section in a rural setting, research studies conducted in an Indian context identifying support or challenge to such a piece of information can be referred to.

Author response: Respected reviewer, thank you for the inputs. We have revised our discussion section by identifying and reporting the studies that support/challenge our findings. With respect to discussion of the finding on high odds of C-section in rural settings, the corresponding change had been made in lines 344-349.

Mechanisms Connecting C-section with HRQL: Elaborate on why C-section is linked to poor HRQL. Could it be attributed to a longer recovery time, pain associated with surgeries, psychosocial effects of such surgeries, or is it because of other variables?

Author response: Thank you for the suggestion. We have strengthened our discussion better elaborating the association of HRQL to C-section. The changes were made to lines 373-374.

Limitations Section: The existing limitations section is very short. It shall be enlarged to cover all issues in relation to:

The basic constraint of cross-sectional design for causal inference.

Recall vulnerability and self-reporting bias in important variables.

The possibility of residual confounding.

Generalizability: Although the sampling in three districts is quite strong, the very high rate of cesarean delivery might not be very representative of all the districts in Tamil Nadu.

Author response: Respected reviewer, thank you for the suggestion. We have improved on our limitations section. The suggested revisions were made in lines 388-401 of the revised file.

4. Minor Corrections and Editorial Suggestions

Abstract: "finical impact" must be replaced with "financial impact".

Introduction: "C-Sections are rapidly raising" should be "rising".

Ways: Add the number of mothers who did not consent out of 510 approached to make it clearer.

Author response: Respected reviewer, thank you for the observations. We have made the suggested changes in the respective sections.

Tables:

Table 1: Make sure the symbol for the conversion in US dollars (^#^) is placed in accordance with the header of the column. Table 1 & 4: Make sure all acronyms are properly explained in a table title or footnote, such as in Table 4 where PCS & MCS in the footnote, but inclusion of PCS & MCS in table 1/4 abbreviation list can help with clarity.

Author response: Respected reviewer, thank you for the observations we have made the necessary edits to present the abbreviations with clarity.

References:

Please make sure all references follow PLOS ONE guidelines. Some references, such as Refs 3, 4, and 9, appear to be web citations or non-traditional sources, and their format can beverified.

Author response: Respected reviewer, thank you for the observations. We have made sure that all references follow PLOS ONE guidelines.

General Proofreading

A general proofreading of this work can help clarify typographical errors in some sentences. For instance, "We conducted this study i)" can be rewritten as "We conducted this study (i)".

Author response: Respected reviewer, thank you for the observation. We have conducted proof reading of the manuscript and made the changes to improve readability of the work.

5. Overall Recommendation Decision: Major Revision. The manuscript considers an important question in research and is well-designed, with important findings presented. However, in order for this manuscript to be publishable, it is important that the above-mentioned issues, especially concerning a proper explanation of methodology limitations, a better analytical presentation, and a better representation of research results, are given proper attention.

Author response: Respected reviewer, thank you for the meticulous review of our manuscript. We have revised our manuscript in accordance with the suggestions made. We believe that we have satisfactorily addressed the comments. We look forward to your positive consideration of our work.

Reviewer 5

Reviewer #5: Caesarean Deliveries and their impact on financial burden and quality of life among

post-partum mothers in Tamil Nadu, India

The study examines a significant public health concern—the rise in Caesarean sections (C-sections) in India—and offers significant empirical evidence on their economic implications and effects on quality of life. Nevertheless, substantial methodological and data-sharing issues need to be resolved prior to its publication.

The C-section rate of 52.6% reported in this study substantially exceeds the WHO recommendation of 10-15%. Examining the factors contributing to and the implications of this trend in Tamil Nadu is significant. Employing the SF-12 survey for quality-of-life evaluation and binary logistic regression for analysis offers a robust quantitative basis.

I would suggest a substantial revision. The comments are presented as follows:

1.Since 60% of mothers delivered within six months prior to the survey and others within up to a year, there is potential for recall bias regarding specific health complications and expenditures. The authors should explicitly address how they mitigated this issue beyond merely "verification with bills," especially concerning the qualitative components of the SF-12 survey.

Author response: We thank the reviewer for this important observation. We acknowledge the possibility of recall bias for self-reported out-of-pocket expenditure and obstetric complications, particularly among women whose delivery occurred up to 12 months prior to the survey. To minimize this, we restricted the recall period to the most recent delivery only and used a structured expenditure schedule with disaggregated cost components. Wherever available, self-reported expenditures were cross-verified with hospital bills and discharge summaries.

The SF-12, however, assesses current health-related quality of life over the preceding four weeks and is not dependent on recall of past pregnancy events; therefore, it is less prone to recall bias. As SF-12 is a self-reported standardized instrument, independent verification is not applicable.

We have now clarified these points in the Methods section and have explicitly acknowledged residual recall bias as a study limitation.

2.The study indicates that mothers residing in rural areas are more likely to have cesarean sections. This contradicts traditional patterns and warrants a more thorough examination of whether it is attributable to insufficient emergency obstetric services or to the practice of "defensive medicine" in rural healthcare facilities.

Author response: Thank you for the observation. While our finding contradict the traditional patterns of urban women more likely to have C-sections, our study is not alone in this. A recent analysis of NFHS-5 on C-sections in south Indian states also observed similar patterns. We reported this and discussed in our discussion section. While we could not specifically examine what are the reasons for higher prevalence of C-sections in rural areas, we recommended the need for future studies to elucidate this.

3.The authors contacted 510 mothers to obtain a final sample of 475, with many exclusions resulting from refusals to provide consent. Including a concise non-response analysis (e.g., comparing the fundamental demographics of individuals who declined participation with those who participated) would assist in demonstrating that your final sample is free from bias.

Author response: Respected reviewer, thank you for the comment. While we agree with the comment, we did not collect any data (including the demographics) from the women who were excluded from the study. Thus we are unable to conduct non-response analysis.

4.Over sixty percent of mothers delivered within six months of the survey, although some were up to a year postpartum. The recall of precise OOPE may diminish over time. The authors confirmed details by cross-referencing hospital bills and discharged summaries in some instances; however, they should explicitly specify the proportion of the sample for which tangible documents were available for verification, compared with those who relied exclusively on recollection.

Author response: Respected reviewer, thank you for the suggestion. While we agree that specifying the proportion of sample for which documents were available for verification will be useful, we were not able to provide the same since we did not explicitly document the availability of hospital bills/discharge summaries in our data collection form.

5.In the logistic regression models, the authors incorporated several independent variables, including district, affluence index, education level, and site of delivery. It is advisable to report Variance Inflation Factors (VIF) or other diagnostic assessments to verify that these variables, particularly wealth and education, are not highly correlated, as such correlation could distort your Adjusted Odds Ratios (AOR).

Author response: Respected reviewer, thank you for the suggestion. We have computed VIF for the independent variables in the model and considered a VIF of < 3.00 as acceptable. We have reported the use of VIF in our methods section.

6.A mother was classified as "high-risk" if she reported at least one indication. However, "prior cesarean section" was a notable indication, accounting for 41.2%. Since a previous C-section almost always leads to a repeat C-section in many clinical settings, it may be beneficial to run a sensitivity analysis excluding these cases to see if the other risk factors (like gestational diabetes or pre-eclampsia) still hold the same weight.

Author response: Respected reviewer, thank you for the suggestion. We have conducted sensitivity analysis by excluding the women who had prior C-section from the analysis. The multivariate models run as part of sensitivity analysis were provided in supplementary file.

7.Quality-of-life scores (SF-12) may vary considerably between one month and twelve months postpartum. It is advisable to incorporate "time since delivery" as a covariate in your regression models for PCS and MCS to ensure that the observed "poor" quality of life is genuinely linked to the mode of delivery rather than merely the recency of childbirth.

Author response: Respected reviewer, thank you for the suggestion. We have revised our multivariate analysis by including the variable “time since delivery” in our multivariate models for PCS and MCS. The revisions were made in Table 6.

________________________________________

6. PLOS authors have the option to publish the peer review history of their article (what does this mean?). If published, this will include your full peer review and any attached files.

If you choose “no”, your identity will remain anonymous but your review may still be made public.

Do you want your identity to be public for this peer review? For information about this choice, including consent withdrawal, please see our Privacy Policy.

Reviewer #1: Yes: Mohsin Hassan Alvi

Reviewer #2: Yes: Montasir Ahmed

Reviewer #3: No

Reviewer #4: No

Reviewer #5: No

Attachment Submitted filename: RSD ROR.docx

# Peer review: Decision Letter 1

**DOI:** 10.1371/journal.pone.0358778.r003

**Authors:** Paridhi Jha

19 Aug 2026

Dear Dr. Kodali,

Please submit your revised manuscript by Oct 03 2026 11:59PM. If you will need more time than this to complete your revisions, please reply to this message or contact the journal office at plosone@plos.org. When you're ready to submit your revision, log on to https://www.editorialmanager.com/pone/ and select the 'Submissions Needing Revision' folder to locate your manuscript file.

- A letter that responds to each point raised by the academic editor and reviewer(s). You should upload this letter as a separate file labeled 'Response to Reviewers'.
- A marked-up copy of your manuscript that highlights changes made to the original version. You should upload this as a separate file labeled 'Revised Manuscript with Track Changes'.
- An unmarked version of your revised paper without tracked changes. You should upload this as a separate file labeled 'Manuscript'.

If you would like to make changes to your financial disclosure, please include your updated statement in your cover letter. Guidelines for resubmitting your figure files are available below the reviewer comments at the end of this letter.

If applicable, we recommend that you deposit your laboratory protocols in protocols.io to enhance the reproducibility of your results. Protocols.io assigns your protocol its own identifier (DOI) so that it can be cited independently in the future. For instructions see: https://journals.plos.org/plosone/s/submission-guidelines#loc-laboratory-protocols. Additionally, PLOS One offers an option for publishing peer-reviewed Lab Protocol articles, which describe protocols hosted on protocols.io. Read more information on sharing protocols at https://plos.org/protocols?utm_medium=editorial-email&utm_source=authorletters&utm_campaign=protocols.

As the corresponding author, your ORCID iD is verified in the submission system and will appear in the published article. PLOS supports the use of ORCID, and we encourage all coauthors to register for an ORCID iD and use it as well. Please encourage your coauthors to verify their ORCID iD within the submission system before final acceptance, as unverified ORCID iDs will not appear in the published article. *Only*  the individual author can complete the verification step; PLOS staff *cannot*  verify ORCID iDs on behalf of authors.

We look forward to receiving your revised manuscript.

Kind regards,

Paridhi Jha, PhD

Academic Editor

PLOS One

Journal Requirements:

1. If the reviewer comments include a recommendation to cite specific previously published works, please review and evaluate these publications to determine whether they are relevant and should be cited. There is no requirement to cite these works unless the editor has indicated otherwise.

2. Please review your reference list to ensure that it is complete and correct. If you have cited papers that have been retracted, please include the rationale for doing so in the manuscript text, or remove these references and replace them with relevant current references. Any changes to the reference list should be mentioned in the rebuttal letter that accompanies your revised manuscript. If you need to cite a retracted article, indicate the article’s retracted status in the References list and also include a citation and full reference for the retraction notice.

[Note: HTML markup is below. Please do not edit.]

Reviewers' comments:

Reviewer's Responses to Questions

**Comments to the Author**

Reviewer #2: All comments have been addressed

Reviewer #5: All comments have been addressed

**********

2. Is the manuscript technically sound, and do the data support the conclusions?

Reviewer #2: Yes

Reviewer #5: Yes

**********

3. Has the statistical analysis been performed appropriately and rigorously?

Reviewer #2: Yes

Reviewer #5: Yes

**********

4. Have the authors made all data underlying the findings in their manuscript fully available?

The PLOS Data policy

Reviewer #2: Yes

Reviewer #5: Yes

**********

5. Is the manuscript presented in an intelligible fashion and written in standard English?

Reviewer #2: Yes

Reviewer #5: Yes

**********

Reviewer #2: Thank you to the authors for thoroughly addressing my previous comments and improving the manuscript considerably. The revised version demonstrates improved methodological clarity, stronger statistical reporting, more cautious interpretation of findings, and a more comprehensive discussion of limitations. I also appreciate the detailed point-by-point responses and the additional analyses conducted in response to the reviewers’ suggestions.

Only a few minor editorial revisions remain. In particular, some residual causal language is still present in the abstract conclusion: “C-section deliveries had a substantial financial impact on the households and reduced the quality of life of post-partum mothers.” Given the cross-sectional nature of the study, this wording may imply causality. It would be more appropriate to revise this as: “C-section deliveries were associated with higher financial burden and poorer quality of life among post-partum mothers.”

Subject to these minor revisions, I recommend acceptance of the manuscript.

Reviewer #5: (No Response)

**********

what does this mean?). If published, this will include your full peer review and any attached files.

If you choose “no”, your identity will remain anonymous but your review may still be made public.

**Do you want your identity to be public for this peer review?**  For information about this choice, including consent withdrawal, please see our Privacy Policy

Reviewer #2: **Yes:** Montasir Ahmed

Reviewer #5: No

**********

[NOTE: If reviewer comments were submitted as an attachment file, they will be attached to this email and accessible via the submission site. Please log into your account, locate the manuscript record, and check for the action link "View Attachments". If this link does not appear, there are no attachment files.]

To ensure your figures meet our technical requirements, please review our figure guidelines: https://journals.plos.org/plosone/s/figures

You may also use PLOS’s free figure tool, NAAS, to help you prepare publication quality figures: https://journals.plos.org/plosone/s/figures#loc-tools-for-figure-preparation.

NAAS will assess whether your figures meet our technical requirements by comparing each figure against our figure specifications.

# Author comment: Author response to Decision Letter 2

**DOI:** 10.1371/journal.pone.0358778.r004

4 Sep 2026

Author response: Respected reviewer, thank you for the suggestion. We agree with the suggestion. We have revised our abstract conclusion as per the revision suggested. The following revision was made to lines 47 and 48 of abstract “C-section deliveries and were associated with higher financial burden and poorer quality of life among post-partum mothers.”

Attachment Submitted filename: ROR_4.9.26.docx

# Editor report: Decision Letter 2

**DOI:** 10.1371/journal.pone.0358778.r005

**Authors:** Paridhi Jha

6 Sep 2026

Caesarean Deliveries and their impact on financial burden and quality of life among post-partum mothers in Tamil Nadu, India

PONE-D-25-51967R2

Dear Dr. Kodali,

We’re pleased to inform you that your manuscript has been judged scientifically suitable for publication and will be formally accepted for publication once it meets all outstanding technical requirements.

Within one week, you’ll receive an e-mail detailing the required amendments. When these have been addressed, you’ll receive a formal acceptance letter and your manuscript will be scheduled for publication.

An invoice will be generated when your article is formally accepted. Please note, if your institution has a publishing partnership with PLOS and your article meets the relevant criteria, all or part of your publication costs will be covered. Please make sure your user information is up-to-date by logging into Editorial Manager at Editorial Manager® and clicking the ‘Update My Information' link at the top of the page. For questions related to billing, please contact billing support.

If your institution or institutions have a press office, please notify them about your upcoming paper to help maximize its impact. If they’ll be preparing press materials, please inform our press team as soon as possible -- no later than 48 hours after receiving the formal acceptance. Your manuscript will remain under strict press embargo until 2 pm Eastern Time on the date of publication. For more information, please contact onepress@plos.org.

Kind regards,

Paridhi Jha, PhD

Academic Editor

PLOS One

Additional Editor Comments (optional):

Reviewers' comments:

# Editor report: Acceptance letter

**DOI:** 10.1371/journal.pone.0358778.r006

**Authors:** Paridhi Jha

PONE-D-25-51967R2

PLOS One

Dear Dr. Kodali,

I'm pleased to inform you that your manuscript has been deemed suitable for publication in PLOS One. Congratulations! Your manuscript is now being handed over to our production team.

At this stage, our production department will prepare your paper for publication. This includes ensuring the following:

* All references, tables, and figures are properly cited

* All relevant supporting information is included in the manuscript submission,

* There are no issues that prevent the paper from being properly typeset

You will receive further instructions from the production team, including instructions on how to review your proof when it is ready. Please keep in mind that we are working through a large volume of accepted articles, so please give us a few days to review your paper and let you know the next and final steps.

Lastly, if your institution or institutions have a press office, please let them know about your upcoming paper now to help maximize its impact. If they'll be preparing press materials, please inform our press team within the next 48 hours. Your manuscript will remain under strict press embargo until 2 pm Eastern Time on the date of publication. For more information, please contact onepress@plos.org.

You will receive an invoice from PLOS for your publication fee after your manuscript has reached the completed accept phase. If you receive an email requesting payment before acceptance or for any other service, this may be a phishing scheme. Learn how to identify phishing emails and protect your accounts at https://explore.plos.org/phishing.

If we can help with anything else, please email us at customercare@plos.org.

Thank you for submitting your work to PLOS One and supporting open access.

Kind regards,

PLOS One Editorial Office Staff

on behalf of

Dr. Paridhi Jha

Academic Editor

PLOS One
